# p66ShcA potentiates the cytotoxic response of triple-negative breast cancers to PARP inhibitors

**DOI:** 10.1172/jci.insight.138382

**Published:** 2021-02-22

**Authors:** Eduardo Cepeda Cañedo, Stephanie Totten, Ryuhjin Ahn, Paul Savage, Deanna MacNeil, Jesse Hudson, Chantal Autexier, Genevieve Deblois, Morag Park, Michael Witcher, Josie Ursini-Siegel

**Affiliations:** 1 Lady Davis Institute for Medical Research, Montreal, Québec, Canada.; 2 Division of Experimental Medicine,; 3 Goodman Cancer Research Centre,; 4 Department of Biochemistry, and; 5 Department of Anatomy and Cell Biology, McGill University, Montreal, Québec, Canada.; 6 Institute for Research in Immunology and Cancer, Montreal, Québec, Canada.; 7 Gerald Bronfman Department of Oncology, McGill University, Montreal, Québec, Canada.

**Keywords:** Cell Biology, Oncology, Adaptor proteins, Apoptosis, Breast cancer

## Abstract

Triple-negative breast cancers (TNBCs) lack effective targeted therapies, and cytotoxic chemotherapies remain the standard of care for this subtype. Owing to their increased genomic instability, poly (ADP-ribose) polymerase (PARP) inhibitors (PARPi) are being tested against TNBCs. In particular, clinical trials are now interrogating the efficacy of PARPi combined with chemotherapies. Intriguingly, while response rates are low, cohort of patients do respond to PARPi in combination with chemotherapies. Moreover, recent studies suggest that an increase in levels of ROS may sensitize cells to PARPi. This represents a therapeutic opportunity, as several chemotherapies, including doxorubicin, function in part by producing ROS. We previously demonstrated that the p66ShcA adaptor protein is variably expressed in TNBCs. We now show that, in response to therapy-induced stress, p66ShcA stimulated ROS production, which, in turn, potentiated the synergy of PARPi in combination with doxorubicin in TNBCs. This p66ShcA-induced sensitivity relied on the accumulation of oxidative damage in TNBCs, rather than genomic instability, to potentiate cell death. These findings suggest that increasing the expression of p66ShcA protein levels in TNBCs represents a rational approach to bolster the synergy between PARPi and doxorubicin.

## Introduction

Triple-negative breast cancers (TNBCs) are a clinically relevant subtype of breast cancer encompassing tumors that lack expression of the estrogen receptor (ER), progesterone receptor, and human epithelial growth factor receptor 2 (HER2) (1, 2). TNBC accounts for approximately 15%–20% of all newly diagnosed breast cancer cases and is associated with poor prognosis (1–4). While many TNBCs are aggressive (5), the standard of care remains chemotherapy, including taxanes and/or anthracyclines, either in the neoadjuvant or adjuvant setting (6). Unlike ER-positive and HER2-positive breast tumors, the poor outcomes of individuals diagnosed with TNBC is mediated, in part, by the lack of effective targeted therapies. Indeed, relapse is commonly observed within the first 5 years after diagnosis. This early relapse is often characterized by the formation of visceral metastases. At relapse, these tumors are generally resistant to the chemotherapies used as standard of care, and the average life expectancy is reduced to less than 18 months (1).

One of the emerging molecular hallmarks that is characteristic of TNBCs is their increased genomic instability. This is in part attributable to the absence of a functional P53 checkpoint, as 65%–80% of TNBCs contain inactivating mutations in the *TP53* gene (7–10). Additionally, the majority of TNBC tumors are considered to have defects in the homologous recombination repair pathway (HR) and are thus referred to as BRCA-like (11–14). The recognition that HR defects reside in a majority of TNBCs, combined with their inherent genomic instability and a lack of targeted therapies, has propelled an interest in testing poly (ADP-ribose) polymerase (PARP) inhibition for this subtype of breast cancer (11, 15).

PARPs are a family of 18 proteins that catalyze the posttranslational modification, poly (ADP-ribosylation) (PARylation) (16, 17), of target proteins. PARP1 is the most highly expressed of the PARP family members, and it has strong catalytic activity. While PARylation is found basally under physiological conditions (18, 19), PARP1 is strongly and rapidly activated in response to DNA damage. This rapid increase in PARylation of target proteins permits the assembly and recruitment of DNA repair complexes (20). HR is required for high-fidelity DNA double-strand break (DSB) repair. Thus, tumors that are defective in HR show acute sensitivity to PARP1 inhibition (21, 22). These studies prompted the development of clinically relevant PARP inhibitors (PARPi) that were initially designed with the intent to block the catalytic activity of PARP1, thereby compromising the capacity of the cell to initiate DNA repair. Through years of building on this concept both preclinically and clinically, PARPi have received FDA approval to treat both breast and ovarian cancers carrying germline *BRCA1/2* mutations. It is now clear that replication fork stress stemming from the trapping of PARP1 on chromatin, especially at sites of genome-embedded ribonucleotides, plays an important role in the antiproliferative effects of PARPi observed in HR compromised cells (23). However, additional mechanisms are likely to contribute to the sensitivity to PARPi. These include promotion of error-prone nonhomologous end-joining repair and the production of ROS (24–29).

The src homology 2 domain–containing gene (*SHC1*) encodes 3 isoforms: p46ShcA, p52ShcA, and p66ShcA. p66ShcA is functionally distinct from the other isoforms (30, 31). p66ShcA is generated through transcriptional initiation from a unique promoter and incorporation of a distinct first exon not found in the other isoforms (32, 33). p46ShcA and p52ShcA act as docking proteins for the transmission of signals downstream of receptor tyrosine kinases (34). However, mitogenic signaling is not a function shared by the longest isoform, p66. Notably, ablation of this isoform contributes to cellular resistance to oxidative stress (30, 35). Under homeostatic conditions, p66ShcA is primarily cytosolic. Upon stress stimuli with oxidants, such as H_2_O_2_ and UV radiation (30, 36), or chemotherapies (37), the serine 36 (S36) residue of p66ShcA is phosphorylated by stress kinases, including p38MAPK, JNK, and PKCβ, allowing it to bind the Pin1 prolyl isomerase and undergo cis/trans isomerization (38). This conformational change allows p66ShcA to translocate into the mitochondria where it promotes the oxidation of cytochrome *c* (cyt c) (39). In this reaction, p66ShcA binding to cyt c facilitates the transfer of electrons from cyt c onto oxygen, leading to ROS production. This requires the cyt c–binding sequence within the protein tyrosine binding domain of p66ShcA (31). Within this sequence, the amino acids E125, E132, E133, W134 and W148 are indispensable for p66ShcA-induced redox activity (40). Increased p66ShcA-mediated ROS production in nontransformed cells leads to disruption of the mitochondrial membrane potential, opening of the permeability transition pore, matrix swelling, disruption of the outer membrane, cyt c release, and apoptosis (41).

Current evidence indicates that ROS production and oxidative stress are important for the cytotoxic effects of commonly used cancer therapies. This includes the cytotoxic activity of PARPi (26–28, 42, 43) and anthracyclines, a common chemotherapy used to treat TNBC (44). Given that p66ShcA is stably overexpressed in a subset of TNBCs (35), we aimed to determine whether p66ShcA-expressing tumors were more sensitive to a combination PARPi/doxorubicin therapy. We hypothesized that high p66ShcA levels in TNBC cells will potentiate cytotoxic levels of ROS, leading to cell death, specifically in response to doxorubicin and PARPi, as both drugs rely on ROS induction as part of their mechanism of action. Here, we validated this concept, demonstrating that increased expression of p66ShcA sensitizes TNBC models to doxorubicin/PARPi combination therapy both in vitro and in vivo as a result of enhanced oxidative stress.

## Results

### Relative p66ShcA levels are not sufficient to predict sensitivity of breast cancer cells to PARPi in combinations therapies.

To test whether p66ShcA levels are predictive of increased chemoresponsiveness in TNBCs, we employed several TNBC patient–derived xenografts (PDXs) that were derived from primary breast cancers before any therapeutic intervention. These PDXs were tested for their relative sensitivity to either doxorubicin (3 mg/kg, i.v. injection, weekly) or cisplatin (4 mg/kg, i.v. injection, weekly) in vivo. Cisplatin is a platinum-based therapy that cross-links DNA, resulting in stalled DNA replication and the indirect induction of DSBs. In contrast, doxorubicin is an anthracycline, which functions as a topoisomerase II inhibitor. Both drugs are known ROS inducers (45, 46). Each PDX was rank ordered based on its relative chemosensitivity and then subsequently interrogated for relative p66ShcA mRNA levels (Supplemental Figure 1; supplemental material available online with this article; https://doi.org/10.1172/jci.insight.138382DS1). We observed a modest association between p66ShcA levels and responsiveness to doxorubicin as well as no correspondence between cisplatin sensitivity and p66ShcA levels, suggesting that p66ShcA-expressing tumors are not inherently more sensitive to chemotherapies. However, the trend toward high p66ShcA levels being associated with responsiveness to doxorubicin in our limited cohort of 7 PDXs prompted us to test whether p66ShcA might act to sensitize breast cancer cell lines to doxorubicin when combined with PARPi. We chose to test niraparib (47), an FDA-approved PARP1/2 inhibitor that is currently under evaluation in breast cancer clinical trials.

### p66ShcA sensitizes TNBC cell lines to doxorubicin/PARPi combination therapies in vitro and in vivo.

**Figure 1:**
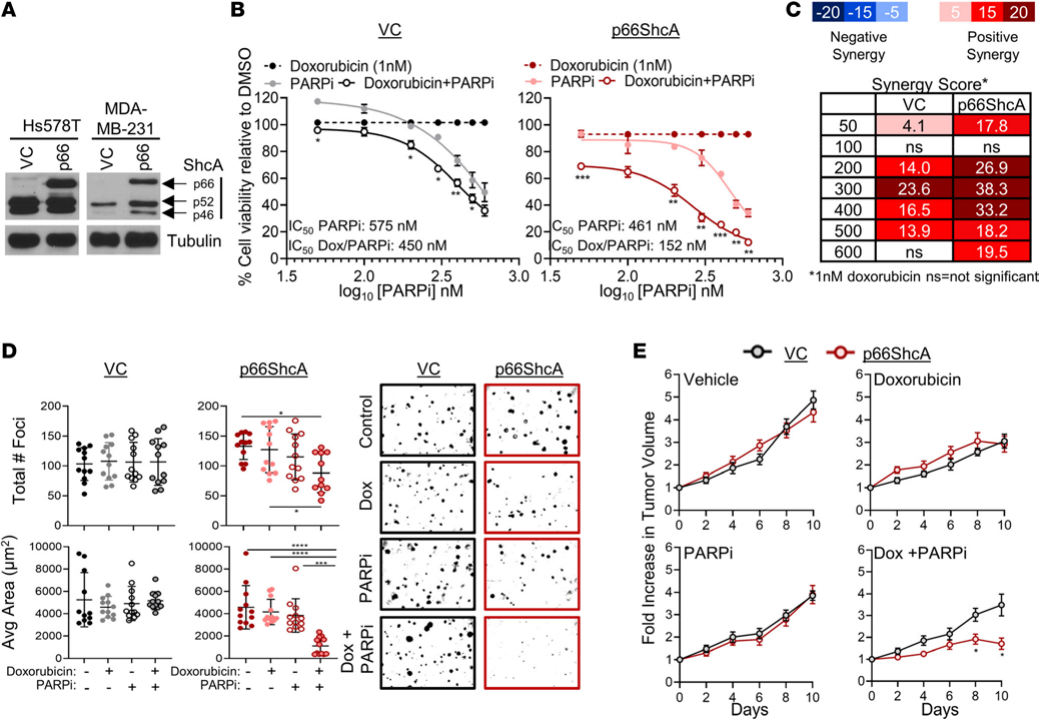
p66ShcA sensitizes TNBC cells to doxorubicin/PARPi combination therapies. (**A**) ShcA and tubulin immunoblot analysis of VC- (p66ShcA null) and p66ShcA-expressing cells. (**B**) Hs578T cells were cultured in DMSO, doxorubicin (1 nM), and PARPi (50–600 nM) alone or in combination for 5 days. Viable cells were quantified by trypan blue exclusion. Data are shown as the mean of mean fold change in cell viability relative to DMSO (mean ± SEM) (*n* = 3 independent experiments). (**C**) Excess-over-Bliss scores. (**D**) Soft agar assay to assess the tumorigenic potential of Hs578T-VC– and p66ShcA-expressing cells cultured in the presence of DMSO, doxorubicin (1 nM), and PARPi (300 nM) alone or in combination for 10 days. Data are representative of 2 independent experiments. The total number of foci and average area of each foci ± SD (*n* = 2 experiments, with 6 technical repeats each) (original magnification, ×40). (**E**) VC- or p66ShcA-expressing Hs578T cells were injected into the mammary fat pads of SCID-Beige mice. At 150mm^3^, mice were randomized into 4 treatment groups: (a) DMSO, (b) 2.5 mg/kg doxorubicin, (c) 25 mg/kg PARPi, or (d) doxorubicin and PARPi combination therapy. Data are shown as fold increase in tumor volume relative to the start of treatment (day 0) ± SEM (*n* = 20–22 tumors per group). Measurements were taken every second or third day following the start of treatment. *P < 0.05; **P < 0.01; ***P < 0.001; ****P < 0.0001 by 2-tailed *t* test (**B**), 1-way ANOVA/Tukey’s multiple comparisons test (**D**), and mixed-effect analysis/Sidak’s multiple comparison tests (**E**).

To investigate whether p66ShcA expression levels might sensitize TNBC to the combination of doxorubicin/PARPi, we generated 2 isogenic model systems using parental cell lines with intermediate levels of p66ShcA. Using CRISPR/Cas9 genomic editing, we deleted endogenous p66ShcA from Hs578T and MDA-MB-231 cells, 2 TNBC cell lines that are *BRCA1/2* WT and express p66ShcA (35). We then reexpressed Flag-tagged WT p66ShcA or alternatively, a corresponding empty vector control (VC) (Figure 1A). We tested the sensitivity of p66ShcA-null (VC) and p66ShcA-positive HS578T or MDA-MB-231 cells to PARPi (50–600 nM) alone or in combination with doxorubicin (1 nM) in a 5-day cell viability assay (Figure 1B and Supplemental Figure 2A). We chose this concentration of doxorubicin as it minimally affected cancer cell viability as a monotherapy (Figure 1B and Supplemental Figure 2A). Both VC- and p66ShcA-expressing cells showed a dose-dependent deleterious effect of increasing PARPi concentrations on cell viability. However, a significantly lower concentration of PARPi was required to achieve 50% inhibition of cell viability in p66ShcA-expressing cells (approximately 400 nM) compared with p66ShcA-null cells (approximately 150 nM) when combined with suboptimal doses of 1 nM doxorubicin (Figure 1B and Supplemental Figure 2A). Excess-over-Bliss analysis (EOB), where scores above 5 are indicative of synergy, revealed that the relationship between PARPi and doxorubicin is indeed synergistic in p66ShcA-expressing cells. Notably, p66ShcA potentiated synergy with doxorubicin at all PARPi concentrations examined (Figure 1C and Supplemental Figure 2B).

Utilizing the drug concentrations that resulted in the highest EOB score in our isogenic lines, we next performed confirmatory viability assays with parental Hs578T and MDA-MB-231 cells compared with their p66ShcA-KO (VC) and p66ShcA-reconstituted counterparts (Supplemental Figure 2C). When used as monotherapies, doxorubicin (1 nM) and PARPi (300 nM) minimally effect cell viability irrespective of p66ShcA status (Supplemental Figure 2D). However, in combination, these therapies lead to an approximately 50% decrease in cell viability, which was less pronounced in p66ShcA-KO (VC) cells, indicating that the combined activity of these drugs is mediated, at least in part, by p66ShcA (Supplemental Figure 2D). Indeed, p66ShcA reexpression significantly sensitized both p66ShcA-null Hs578T and MDA-MB-231 cells to the doxorubicin/PARPi combination therapy, reducing cell viability by 50% and 65% after 3 or 5 days of treatment, respectively (Supplemental Figure 2E). The increased sensitivity of TNBC cells ectopically reexpressing p66ShcA, relative to parental controls, likely reflects increased p66ShcA expression levels (Supplemental Figure 2C).

To extend these results further, we tested whether increasing p66ShcA in TNBC cell lines with low-to-moderate expression levels could potentiate the cytotoxic effects mediated by doxorubicin and PARPi. First, we transduced a p66ShcA-expressing vector (p66ShcA-OE) or the corresponding VC in luminal (MCF7) or triple-negative (Hs578T, MDA-MB-468) breast cancer cell lines (Supplemental Figure 3A). These cell lines were treated with PARPi (100 or 300 nM) and doxorubicin (1 or 2 nM) alone or in combination for 5 days. Whereas increased p66ShcA levels had no effect on cell viability when doxorubicin and PARPi were used as monotherapies, p66ShcA overexpression sensitized both TNBC cell lines to doxorubicin/PARPi combination therapy. In contrast, MCF7 cell viability to this same combination therapy was p66ShcA independent.

We next probed whether endogenous p66ShcA levels were sufficient to predict sensitivity to doxorubicin/PARPi combination treatment in a similar manner to our isogenic lines. This included p66ShcA-deficient models of human luminal (MCF7), HER2-positive (HCC1954) and triple-negative (MDA-MB-468) breast cancer as well as p66ShcA-positive TNBC lines (Hs578T, MDA-MB-231, BT549, BT20) (Supplemental Figure 4A). We tested the sensitivity of each cell line to PARPi (50–600 nM) alone or in combination with doxorubicin (1 or 2 nM) in a 5-day cell viability assay. Endogenous p66ShcA levels were not sufficient to predict absolute sensitivity to doxorubicin/PARPi combination therapy (Supplemental Figure 4, B and C). However, based on EOB scores, p66ShcA positivity in TNBCs does predict stronger synergy between doxorubicin and multiple PARPi concentrations, consistent with data generated from isogenic lines (Supplemental Figure 4B). In contrast, p66ShcA-negative breast tumors showed little evidence of synergy between doxorubicin and PARPi treatment, irrespective of breast cancer subtype (Supplemental Figure 4C). Our experiments thus far focused on combining doxorubicin with PARPi. Given that cisplatin is a clinically relevant combination with PARPi (48, 49), we wanted to test whether p66ShcA could also predict sensitivity to cisplatin in combination with PARPi. For this, parental, p66ShcA-KO, and p66ShcA-reconstituted Hs578T cells were exposed to 300 nM PARPi and 100 nM cisplatin alone or in combination over a period of 3 and 5 days (Supplemental Figure 5). As suggested by our PDX studies (Supplemental Figure 1), p66ShcA status did not predict sensitivity to cisplatin either alone or in combination with PARPi. This indicates that doxorubicin acts through mechanisms distinct from cisplatin, enhancing its activity when combined with PARPi in p66ShcA-expressing cells.

We next assessed whether the sensitivity of TNBCs to doxorubicin/PARPi treatment was also p66ShcA dependent when cells were cultured under anchorage-independent growth conditions. Again, suboptimal doses of doxorubicin or PARPi, either as monotherapies or in combination, did not have a measurable effect on foci formation for p66ShcA-null (VC) Hs578T cells (Figure 1D). In contrast, p66ShcA expression led to a 40% reduction in foci formation when both drugs were combined (Figure 1D). More strikingly, p66ShcA expression was absolutely required for doxorubicin/PARPi combination treatment to abrogate their continued growth, with a 4-fold reduction of foci area (Figure 1D). Together, these results strongly suggest that p66ShcA sensitizes TNBC cells to doxorubicin/PARPi combination therapy in vitro.

We further examined the important possibility that p66ShcA sensitizes TNBCs to doxorubicin/PARPi treatment in vivo. Consistent with our in vitro studies, p66ShcA-null (VC) or p66ShcA-expressing breast tumors were relatively insensitive to the tumoricidal properties of doxorubicin and PARPi as monotherapies. In contrast, at endpoint, p66ShcA-expressing breast tumors displayed a striking, 5-fold reduction in growth following doxorubicin/PARPi combination treatment compared with that in p66ShcA-null tumors (Figure 1E and Supplemental Figure 2E). In contrast, the growth rate of p66ShcA-null tumors was only decreased by 1.6-fold in response to this combination treatment (Figure 1E and Supplemental Figure 2E). Furthermore, whereas the p66ShcA-null breast tumors showed sustained growth following exposure to doxorubicin/PARPi, a “stable disease” phenotype was observed in p66ShcA-expressing tumors (Figure 1E and Supplemental Figure 2E). Altogether, these data suggest that p66ShcA sensitizes TNBCs to doxorubicin/PARPi combination therapy in a synergistic manner.

### p66ShcA exerts a cytotoxic effect in TNBCs in response to doxorubicin/PARPi treatment.

We next sought to determine the molecular basis for the ability of p66ShcA to sensitize TNBCs to doxorubicin/PARPi combination therapy, both in vitro and in vivo. Given the established role for PARPi in mediating a G_2_/M arrest in response to replicative stress (50), we first examined whether p66ShcA altered the cell cycle distribution of TNBCs treated with doxorubicin/PARPi using flow cytometric propidium iodide (PI) staining. Although we observed a mild accumulation of cells in the G_2_/M phase concomitant with a reduced distribution in the G_1_ phase of the cell cycle following 48-hour treatment with doxorubicin/PARPi, these differences were independent of p66ShcA levels (Supplemental Figure 6A). We also did not observe profound differences in the number of Ki67-positive cancer cells in vivo in response to doxorubicin/PARPi treatment, both in p66ShcA-null and p66ShcA-expressing Hs578T tumors (Supplemental Figure 6C). These data suggest that p66ShcA-induced inhibition of breast cancer cell viability and tumor growth in response to doxorubicin/PARPi was not due to cell cycle alterations. These data led us to examine whether p66ShcA expression increased the ability of doxorubicin/PARPi combination treatment to stimulate apoptosis in TNBCs.

**Figure 2:**
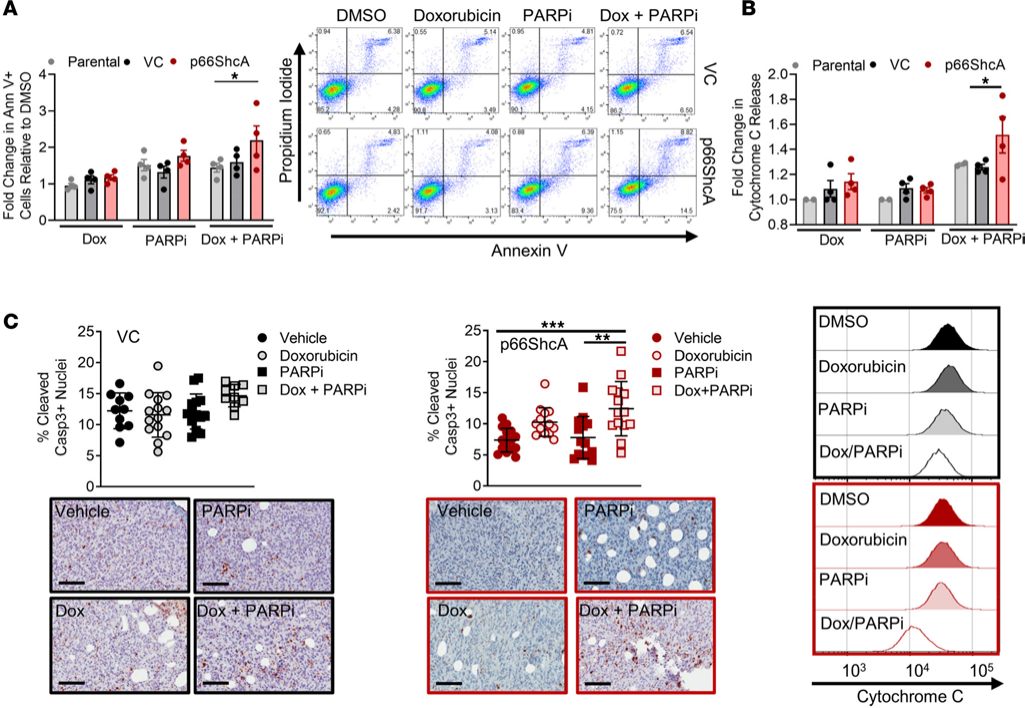
p66ShcA increases apoptosis induced by doxorubicin/PARPi in a TNBC model. Parental, p66ShcA-null (VC), or p66ShcA-reconstituted Hs578T cells were treated with DMSO, doxorubicin (1 or 10 nM), and PARPi (300 nM), alone or in combination, and assessed by (**A**) annexin V staining at 72 hours or (**B**) cytochrome *c* release at 48 hours. (**A**) Results are presented as average fold change in percentage of annexin V–positive cells relative to DMSO ± SEM (*n* = 4 independent experiments). (**B**) Results are presented as average fold change percentage of cytochrome *c* release relative to DMSO ± SEM (*n* = 2–4 independent experiments). (**C**) VC- and p66ShcA-expressing Hs578T tumors were treated with doxorubicin alone (2.5 mg/kg), PARPi alone (25 mg/kg), doxorubicin and PARPi in combination, or DMSO. Apoptosis was assessed by cleaved caspase-3 IHC. Data are depicted as percentage (average ± SEM) of positive cleaved caspase-3 cells (*n* = 10–12 tumors per group), and representative images are shown (scale bars: 100 μm). *P < 0.05; **P < 0.01; ***P < 0.001 by 2-way ANOVA/Tukey’s multiple comparisons test (**A** and **B**) and 1-way ANOVA/Tukey’s multiple comparisons test (**C**).

In response to stress stimuli, mitochondrial p66ShcA has been shown to disrupt the mitochondrial membrane potential, leading to opening of the permeability transition pore, mitochondrial matrix swelling, disruption of the outer member, cyt c release, and apoptosis (31, 41). Indeed, annexin V flow cytometric analysis revealed that high p66ShcA levels in Hs578T cells resulted in an approximately 60% increase in the apoptotic rate of breast cancer cells in response to doxorubicin/PARPi treatment compared with that in p66ShcA-null cells and the parental line, which expressed lower endogenous p66ShcA levels (Figure 2A and Supplemental Figure 2C). Consistent with this increased apoptotic index, doxorubicin/PARPi treatment led to a 25% increase in cyt c release in p66ShcA-high expressing Hs578T cells compared with that in VC and parental cells (Figure 2B). To validate our results in vivo, we performed cleaved caspase-3 immunohistochemical (IHC) staining of Hs578T breast tumors treated with doxorubicin and PARPi, either as monotherapies or in combination (Figure 1E). Interestingly, p66ShcA-expressing breast tumors had a reduced basal apoptotic rate compared with that in p66ShcA-null tumors (7.5% versus 12.5%), suggesting that p66ShcA may actually be cytoprotective under steady-state conditions (Figure 2C). However, following combined doxorubicin and PARPi treatment, we observed a 65% increase in the apoptotic rate of p66ShcA-expressing tumors compared with an 20% increase observed in the p66ShcA-deficient controls (Figure 2C). In contrast, neither doxorubicin nor PARPi as monotherapies appreciably altered the apoptotic potential of p66ShcA-null or p66ShcA-expressing tumors at the drug concentrations used in this study (Figure 2C). Thus, in response to severe and acute therapy-induced stress, p66ShcA may increase the cytotoxic potential of doxorubicin/PARPi exposure in TNBCs partly by stimulating cyt c release and apoptosis. However, p66ShcA expression only induced a modest increase in apoptosis of MDA-MB-231 cells following combined drug treatment (Supplemental Figure 7). In contrast, we observe a moderate but statistically significant increase in the G_2_/M cell cycle arrest in p66ShcA-expressing MDA-MB-231 cells that was not observed in VCs (Supplemental Figure 6B). These data suggest that the antitumor effects exerted by p66ShcA may be context dependent and affect cell cycle arrest and/or programmed cell death or alternatively function through distinct mechanisms controlling cell viability.

### The ability of p66ShcA to enhance the antitumor effects of doxorubicin/PARPi combination therapy involves mechanisms beyond the accumulation of DNA damage.

**Figure 3:**
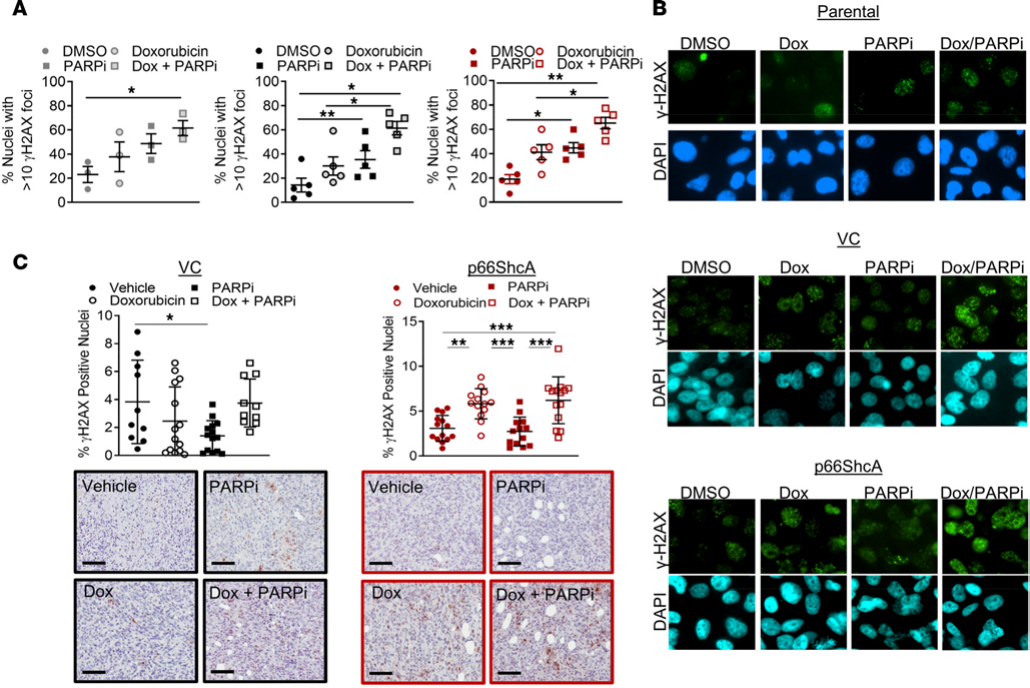
The DNA damage response is unaffected by p66ShcA in response to doxorubicin/PARPi combination therapy. (**A**) Parental, VC- (p66ShcA-null), and p66ShcA-expressing Hs578T cells were treated with doxorubicin (1 nM) and PARPi (300 nM) alone or in combination for 48 hours. Double-strand DNA breaks were assessed by γH2AX immunofluorescence staining. Percentage nuclei with >10 γH2AX foci was quantified ± SEM (*n* = 3–5 independent experiments). (**B**) Representative images are shown (original magnification, ×400). (**C**) VC- and p66ShcA-expressing Hs578T tumors were treated with doxorubicin alone, PARPi alone, doxorubicin and PARPi in combination, or vehicle control. DNA damage levels were determined by γH2AX IHC staining. Data are depicted as (average ± SEM) percentage of positive γH2AX nuclei (*n* = 10–12 tumors per group). Representative images IHC staining illustrating γH2AX-positive nuclei are shown (scale bars: 100 μm). *P < 0.05; **P < 0.01; ***P < 0.001 by 1-way ANOVA/Tukey’s multiple comparisons test.

Previous reports suggest that the effectiveness of PARPi in combination with chemotherapies is associated with the deleterious accumulation of DNA damage (51, 52). Therefore, we aimed to determine whether the antitumor effects observed after doxorubicin/PARPi exposure were associated with an accumulation of cytotoxic DNA DSBs in p66ShcA-expressing cells. To test this, we probed for S139-phosphorylated H2AX (γH2AX) using immunofluorescence staining in vehicle- and drug-treated VC- and p66ShcA-expressing cells. Once DSBs are generated, large tracts of surrounding chromatin are decorated with γH2AX, giving the appearance of distinct foci using fluorescence microscopy (53). TNBC cells are genomically unstable and must cope with high intrinsic levels of DNA damage. Thus, it is not surprising that the increase in DNA damage accrued because of doxorubicin or PARPi alone (approximately 40% γH2AX-positive staining in parental, VC, and p66ShcA cells) did not lead to proportional decreases in cell viability (Figure 3A and Supplemental Figure 8). As expected, the combination of doxorubicin/PARPi resulted in a large increase in the accumulation of DSBs, but this effect was independent of p66ShcA expression. Specifically, the proportion of nuclei with more than 10 foci after the doxorubicin/PARPi exposure was approximately 60% for both Hs578T (parental, VC, and p66ShcA reconstituted) and MDA-MD-231 (VC and p66ShcA reconstituted) cells (Figure 3A and Supplemental Figure 8).

We additionally assessed DNA damage within control- and drug-treated Hs578T tumors (Figure 1E) through γH2AX IHC staining. In p66ShcA-null (VC) tumors, DNA damage was unaffected by treatment with doxorubicin and PARPi, alone or in combination (Figure 3B). Although p66ShcA potentiated the accumulation of DSBs in tumors following doxorubicin treatment, it did not significantly inhibit the growth rate of these tumors when treated with doxorubicin as a monotherapy (Figure 1E and Figure 3B). Moreover, PARPi treatment alone had no effect on the accumulation of DSBs in p66ShcA-expressing tumors. Finally, the number of γH2AX-positive nuclei observed upon exposure to doxorubicin/PARPi mirrored that observed with exposure to doxorubicin alone (3% to 6% positive nuclei) and is unlikely to account for the 5-fold decrease in tumor volume specifically observed in doxorubicin/PARPi-treated, p66ShcA-expressing tumors (Figure 1E and Figure 3C). Together, these data suggest that the sensitization of TNBC cells to doxorubicin/PARPi treatment conferred by p66ShcA is dependent on additional factors beyond the accumulation of DNA damage.

### The ability of p66ShcA to enhance the antitumor effects of doxorubicin/PARPi combination therapy involves mechanisms beyond metabolic stress.

Another plausible explanation for the cooperation seen between doxorubicin and PARPi in p66ShcA-expressing cells is susceptibility to metabolic stress. p66ShcA is a multifunctional protein, and its mitochondrial function is not only related to ROS production, but also the promotion of a catabolic state (54). Likewise, both doxorubicin and PARPi have marked effects on cellular respiration and metabolic processes. Doxorubicin increases de novo pyrimidine synthesis (55) and is known to repress mitochondrial respiration at high concentrations (56). PARPi may alter survival and the balance between oxidative phosphorylation and glycolytic pathways through shifts in NAD+ consumption (57). We hypothesized that high p66ShcA levels might exacerbate metabolic stress following doxorubicin/PARPi exposure. In contrast, we observed similar basal oxygen consumption rates (OCRs) between VC- and p66ShcA-expressing cells. Upon exposure to suboptimal doses of PARPi and doxorubicin, neither agent alone affected cellular respiration. In VC cells, the combination therapy modestly reduced both the basal respiratory rate and maximal respiratory capacity of the cells. However, in p66ShcA-expressing cells, these changes were not apparent (Supplemental Figure 9A). Moreover, IHC analysis of p66ShcA-deficient or -proficient breast tumors did not show any differences in pAMPK and pACC levels (both markers of energetic stress) when treated with doxorubicin or PARPi, either as monotherapies or in combination (Supplemental Figure 9, B and C). Thus, changes in cellular respiration cannot account for the increased sensitivity of p66ShcA-expressing tumors to doxorubicin/PARPi treatment.

### p66ShcA-induced oxidative stress sensitizes TNBCs to doxorubicin/PARPi therapy.

**Figure 4:**
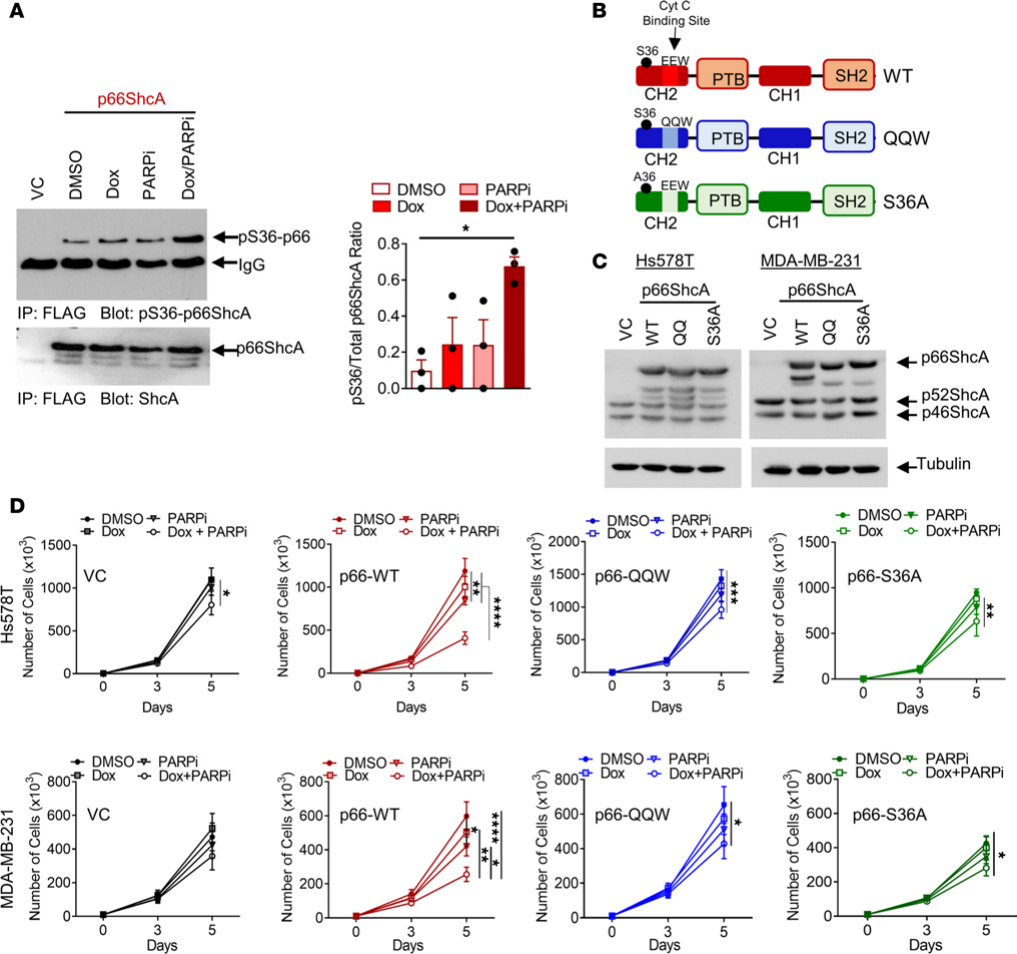
p66ShcA-induced oxidative stress sensitizes TNBCs to doxorubicin/PARPi. (**A**) Hs578T cells were cultured in the absence (DMSO) or presence of doxorubicin (10 nM) or PARPi (300 nM) alone or in combination for 48 hours. FLAG immunoprecipitates were blotted with pS36-p66ShcA or ShcA antibodies. Data are shown as average ratio of pS36-p66ShcA/p66ShcA ± SEM (*n* = 3 independent experiments). (**B**) Schematic diagram depicting p66ShcA-WT, p66-QQW, or p66-S36A mutants. (**C**) Immunoblot showing relative p66ShcA expression levels. (**D**) VC-, p66-WT–, p66-QQW–, or p66-S36A–expressing cells were treated with doxorubicin (1 nM) and PARPi (300 nM), alone or in combination. The number of viable cells was determined by trypan blue exclusion. The graphs show the average number of cells ± SEM (*n* = 3–6 independent experiments). *P < 0.05; **P < 0.01; ***P < 0.001; ****P < 0.0001 by 1-way ANOVA/Tukey’s multiple comparisons test (**A**) and 2-way ANOVA/Tukey’s multiple comparisons test (**D**).

Previous studies have shown that S36 phosphorylation of p66ShcA potentiates its proapoptotic activity by facilitating the production of ROS (31). Thus, we postulated that doxorubicin and/or PARPi treatment could increase p66ShcA S36 phosphorylation in TNBCs. Although S36 phosphorylation of p66ShcA was only marginally increased with doxorubicin or PARPi when used as monotherapies, treatment of TNBCs with this combination therapy significantly increased p66ShcA S36 phosphorylation levels (3-fold) beyond what was observed with either drug as a monotherapy (Figure 4A). This synergistic induction of S36 p66ShcA phosphorylation suggested that the molecular mechanism underlying p66ShcA sensitization to doxorubicin/PARPi therapy may be through enhanced generation of deleterious mitochondrial ROS.

The highly reactive and unstable nature of ROS makes it challenging to reliably measure differences in steady-state ROS levels over a prolonged period. Therefore, we combined genetic approaches, pharmacological ROS scavenger studies, and the accumulation of oxidative DNA damage to accurately portray the contribution of p66ShcA-induced mitochondrial ROS on increased sensitivity to doxorubicin/PARPi treatment. First, we reconstituted p66ShcA-null Hs578T and MDA-MB-231 cells with a p66ShcA mutant allele harboring point mutations in the cyt c–binding site (amino acids E132 and E133 to QQ) (herein referred to as p66ShcA-QQ) or the S36 phosphorylation site (referred as p66ShcA-S36A) (Figure 4, B and C). Although p66ShcA-QQ retains the ability to localize to the mitochondria in response to stress stimuli, it lacks critical glutamic acid residues that mediate its interaction with cyt c, thereby preventing the formation of ROS upon stress (31). On the other hand, the p66ShcA-S36A mutant lacks the crucial phosphorylation site that mediates its translocation into the mitochondria (58). We carried out 5-day viability assays via trypan blue exclusion to assess the growth rate of TNBC cell lines that lacked p66ShcA (VC) or expressed the p66ShcA-WT, p66ShcA-QQ, or p66ShcA-S36A proteins (Figure 4D). Consistent with our previous data (Figure 1B and Supplemental Figure 5D), WT p66ShcA-expressing cells showed considerable sensitivity to the combination of doxorubicin/PARPi. Importantly, this synergy was ablated in cells harboring both the p66ShcA-QQ and p66ShcA-S36A mutants (Figure 4D). These data strongly suggest that the translocation of p66ShcA to the mitochondria followed by its interaction with cyt c and subsequent ROS production is critical for the sensitization of p66ShcA cells to the combination therapy.

**Figure 5:**
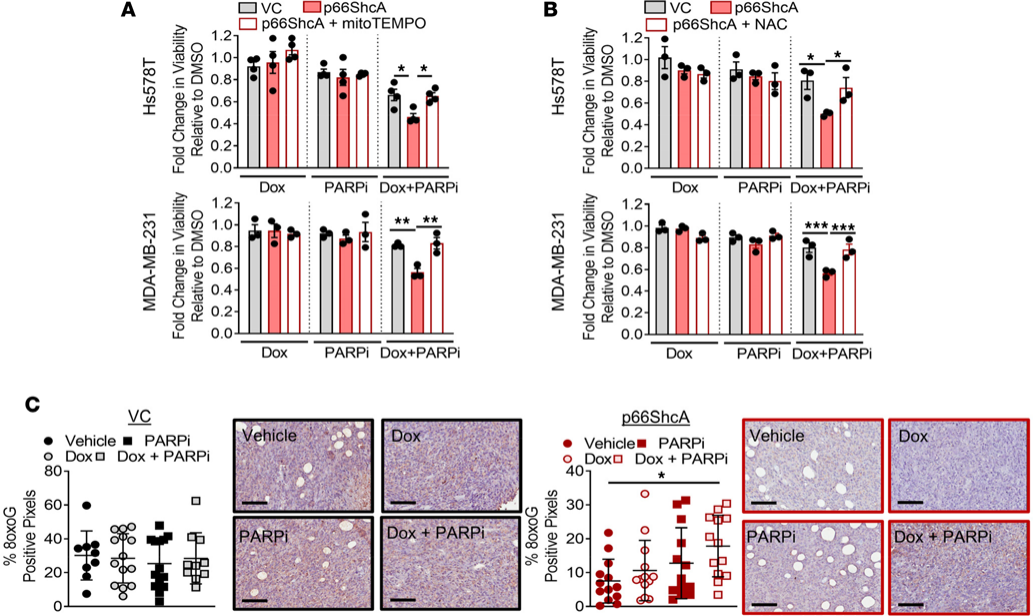
ROS scavengers reverse p66ShcA-induced sensitivity of TNBCs to doxorubicin/PARPi combination therapy. VC- and p66ShcA-expressing Hs578T and MDA-MB-231 cells were treated with doxorubicin (1 nM) and PARPi (300 nM) alone or in combination, in presence or absence of (**A**) MitoTEMPO (10 μM) or (**B**) NAC (5 mM). Cell viability was determined by trypan blue exclusion. Data are shown as the mean of mean fold change of the number of viable cells relative to DMSO (mean ± SEM) (*n* = 3–4 independent experiments). (**C**) Oxidative stress marker 8-oxodG was analyzed by IHC staining in VC- and p66ShcA-expressing Hs578T tumors. Data are depicted as average percentage of 8-oxodG–positive pixels ± SEM (*n* = 10–12 tumors per group). Representative images of the IHC staining illustrating 8-oxodG positivity are shown (scale bars: 100 μm). *P < 0.05; **P < 0.01; ***P < 0.001 by 2-way ANOVA/Tukey’s multiple comparisons test (**A** and **B**) and 1-way ANOVA/Tukey’s multiple comparisons test (**C**).

To further examine whether ROS production was required to induce p66ShcA-induced synergy of TNBC cells to doxorubicin/PARPi treatment, cell viability assays were carried out in the presence of ROS scavengers, including mitoTEMPO, a mitochondrial superoxide anion scavenger, or N-acetyl-cysteine (NAC), a precursor for glutathione synthesis. ROS scavengers were administered to the cells 24 hours before drug exposure and then replenished daily. Both scavengers were able to rescue the viability of p66ShcA-expressing cells after 3 and 5 days of doxorubicin/PARPi treatment to the levels of drug-treated VC cells (Figure 5, A and B, and Supplemental Figure 10, A and B). Although p66ShcA-null (VC) tumors had higher basal 8-oxodG levels, a marker of oxidative DNA damage, they were unaffected by doxorubicin or PARPi, alone or in combination (Figure 5C). In contrast, p66ShcA tumors showed increased 8-oxodG accumulation when treated with doxorubicin and PARPi, likely reflecting increased oxidative damage of mitochondrial DNA. Overall, these data strongly support the hypothesis that p66ShcA generates mitochondrial ROS to sensitize TNBCs to doxorubicin/PARPi combination therapy.

## Discussion

The advent of PARPi represented a promising targeted therapy for individuals with TNBC. The recognition that many TNBCs carry defects in HR, referred to as BRCAness, led researchers to explore PARPi as monotherapies for this subtype. Early clinical trials showed a lack of objective clinical responses in women with sporadic TNBC compared with those with *BRCA1/2* mutated breasts cancers, who most benefited from this targeted therapy (59, 60). Although the results from these early-stage clinical trials seemed promising for individuals with TNBC *BRCA1/2* mutant disease, the stage III clinical trial OlympiAD showed no benefit of adding PARPi to the standard of care for advanced breast cancers (61). Subsequent and ongoing clinical trials have tested the efficacy and cytotoxic effects of standard-of-care chemotherapies with PARPi for metastatic TNBC and as neoadjuvant therapies (62). Notably, a large TNBC clinical trial found that the PARPi veliparib when added to carboplatin and paclitaxel in a neoadjuvant setting, potentiated pathological complete responses when doxorubicin and cyclophosphamide were applied as adjuvant therapies (63). This study underscores the potential of PARPi as a combination therapy.

Currently, *BRCA1/2* mutations and HR defects are the primary predictors of response to PARPi. However, studies suggest that response to PARPi can be bolstered only for those tumors staining positive for the primary target of PARP1 (64, 65). HR-defective tumors show sensitivity to PARPi through multiple mechanisms. These include promotion of DNA repair through nonhomologous end joining that, over time, leads to deleterious genomic errors and replication fork stress stemming from the trapping of PARP1 on chromatin, especially at sites of genome-embedded ribonucleotides. Recent studies suggest additional mechanisms behind the cytotoxicity exerted by PARPi in cancer cells involving ROS production (26–29, 42, 43). Given that p66ShcA, a known ROS inducer, is enriched in highly metastatic breast cancer models and TNBC cell lines (35, 58, 66), we investigated whether p66ShcA could sensitize TNBCs to the combination of PARPi with chemotherapy. Although our data do not support endogenous p66ShcA levels as a biomarker for sensitivity to doxorubicin/PARPi combination therapy, our data strongly indicate that boosting p66ShcA expression levels synergistically increases responsiveness of TNBCs to doxorubicin/PARPi through increased oxidative stress. In BRCA1/2-deficient cells, the efficacy of PARP inhibition is mediated by trapping PARP1/2 on chromatin, leading to stalled replication forks. Our data, in keeping with previous reports (28), indicate that, in cells beyond those associated with a BRCAness phenotype, PARPi potentiate cell death through enhanced ROS production. This was clearly demonstrated through the use of scavengers that partially ameliorated the loss of proliferation after exposure to PARPi. While other reports suggest that such ROS promotes genomic instability (28), we did not find DSB to correlate with cell death in our models. Thus, we propose that, in p66ShcA-elevated tumors, the loss of proliferation mediated by PARP trapping to chromatin is secondary to their capacity to elevate cytotoxic ROS. We propose that it worthwhile to compare ROS elevation after exposure to a spectrum of clinically relevant PARPi in combination with suboptimal doses of doxorubicin. Integrating measurements of cell death, oxidative stress markers, and trapping capacity could inform on the optimal PARPi to employ in p66ShcA-expressing cells, and the relative contribution of ROS production, or trapping, in mediating a cytotoxic response.

Clinical data on the combination of doxorubicin with PARPi in TNBC is scarce. The utilization of doxorubicin as one of the first lines of treatment in TNBC (67, 68) limits the use of this anthracycline in combination with PARPi in advanced disease due to acquired resistance. Interestingly, cells with the highest p66ShcA levels were not as sensitive to the combination therapy. This may be due, in part, to the role of p66ShcA in promoting plasticity and resistance to chemotherapy (35), along with potential adaptive responses to sustained oxidative stress in tumors that must cope with chronically elevated p66ShcA levels.

Nevertheless, doxorubicin/PARPi combinations could be administered as part of the adjuvant or neoadjuvant regime in patients with moderately elevated p66ShcA-expressing TNBC. Furthermore, given that the expression of p66ShcA is regulated by promoter methylation, the use of epigenetic drugs, such as DNA methyltransferase inhibitors, to increase its levels followed by doxorubicin/PARPi could be an interesting combination strategy to explore. Indeed, a preclinical study showed that the PARPi talazoparib exquisitely synergized with guadecitabine, proving to be an effective combination therapy to treat breast and ovarian cancers irrespective of their *BRCA1/2* status. Furthermore, the cytotoxic activity of this combination was shown to be mediated by ROS (26). Whether p66ShcA is a key mediator of the antitumor effects caused by demethylating agents and PARPi combination remains to be explored (69, 70).

Another practical finding of our study is that the effective dose of doxorubicin could be lowered when used in combination with PARPi for those tumors with moderate-to-high expression of p66ShcA. This may ameliorate the deleterious side effects of anthracyclines on cardiotoxicity while conserving their antitumor activity. Furthermore, there is preclinical evidence of cardiomyocyte protection upon administration of PARPi (71). Whether the administration of PARPi along with anthracyclines would ameliorate the side effects of the chemotherapy or exacerbate them should be tested yet is beyond the scope of this study.

Mechanistically, we confirmed that doxorubicin and PARPi, when used at suboptimal doses and as monotherapies, did not appreciably alter the redox potential of p66ShcA. However, the combination of doxorubicin/PARPi at the same concentrations worked in synergy to significantly activate this redox protein. Moreover, the ability of p66ShcA to stimulate mitochondrial ROS production is required to sensitize TNBCs to this drug combination. Furthermore, our data suggest that this response is in part dependent on the induction of apoptosis, stemming from the production of ROS. Indeed, a proapoptotic effect of PARPi in combination with doxorubicin in *TP53* mutated breast cancer cell lines has been previously shown to be linked to the loss mitochondrial membrane potential and cyt c release (72). Although we demonstrated that there is a significant increase in DSBs upon treatment with doxorubicin/PARPi, the observed spike was exclusively dependent on drug activity and was not influenced by the expression of p66ShcA in TNBC cell lines. These data on DNA DSBs does not correlate with our data showing a p66ShcA-dependent enhanced reduction in viability upon doxorubicin/PARPi treatment, both in vitro and in vivo. p66ShcA-expressing tumors showed significant induction of DSBs upon treatment with doxorubicin alone, but this did not lead to a considerable repression of tumor volume over the time period examined.

As described by Ott et al. (73), the release of cyt c and initiation of apoptosis, requires (a) the solubilization of cyt c in the intermembrane space of the mitochondria by its detachment from cardiolipin and (b) the formation of a pore in the mitochondrial outer membrane. Furthermore, cyt c–mediated peroxidation of cardiolipin contributes to its solubilization (74, 75). It is possible that the activation of p66ShcA and translocation into the mitochondria intermembrane space primes the initiation of apoptosis by potentiating the oxidation of cardiolipin. Concomitant with p66ShcA production of ROS, the DSBs generated in the nuclei by the combination therapy will further potentiate proapoptotic signaling.

Preclinical studies have established that PARP inhibition enhances the cytotoxic activity of doxorubicin by promoting the accumulation of DNA damage (72, 76, 77). It should be considered that the concentrations of doxorubicin used in these studies are on average one thousand–fold higher than the levels employed in our study. Thus, increasing p66ShcA protein levels may allow for a reduction of the amount of doxorubicin used and, thus, ameliorate its unwanted cytotoxic effects.

## Methods

### Cell culture.

Hs578T, MDA-MB-231, BT20, BT549, MDA-MB-468, and MCF7 cells were obtained from ATCC and cultured in 10% FBS (Wisent Bio Products, catalog 080-150) DMEM (Wisent Bio Products, catalog 319-005-CL). HCC1954 cells were cultured in 10% FBS RPMI 1640 media (Wisent Bio Products, catalog 350-000-EL). All cell lines were routinely screened for mycoplasma contamination using MycoAlert (Lonza, catalog LT07-118) at least once per month or 1 day before mammary fat pad injection.

### CRISPR/Cas9 genome editing.

p66ShcA was deleted from the genome of Hs578T and MDA-MB-231 cells using CRISPR/Cas9 genomic editing (p66ShcA-KO). Sequences targeting p66ShcA at its CH2 domain were determined by the CRISPR Design Tool (https://zlab.bio/guide-design-resources): 5′-GAGGCTGGCCAACCCGGCTGGGG-3′. p66ShcA-deficient cells were generated as previously described (35) and were selected with 10 μg/mL blasticidin (Wisent Bio Products, catalog 400-190-EM). p66ShcA-KO cell lines represent pools of 5 clones.

C-terminal flag-tagged mouse p66ShcA-WT, p66ShcA-QQ mutant (31), and p66ShcA-S36A mutant (58) were subcloned into the pMSCV/Puromycin expression vector (Clontech, catalog 68469) using XhoI and EcoRI restriction sites. VC, flag-tagged p66ShcA-WT, p66ShcA-QQ, and p66ShcA-S36A mutant constructs were transfected into a retroviral packaging cell line, Phoenix (293T), using Effectene Transfection Reagent (Qiagen, catalog 301425) as per the manufacturer’s protocol. Filtered viral supernatants were used to infect Hs578T and MDA-MB-231 p66ShcA-KO cell lines. Additionally, VC and p66ShcA-WT carrying pMSCV vectors were virally transduced into Hs578T and MDA-MB-468 parental cell lines following the same protocol. The MCF7 parental cell line was transduced with viral supernatants of VC or p66ShcA-WT cloned into the NotI and EcoRI restriction sites of PQCXIP vector (Clontech, catalog 631516). Cell lines were selected with 2 μg/mL puromycin.

### Inhibitors and ROS scavengers.

Cell lines were treated with media carrying DMSO (BioShop, catalog DMS666) as a control, doxorubicin (1–2 nM; MilliporeSigma, catalog D1515), cisplatin (100 nM; TOCRIS, catalog 2251), and PARPi MK-4827 (300 nM; APExBIO, catalog A3617) alone or in combination (doxorubicin/PARPi). Where indicated, cells were treated with ROS scavengers, mitoTEMPO (10 μM; MilliporeSigma, catalog SML0737) or pH 7.4 buffered NAC (5 mM; MilliporeSigma, catalog A9165).

### Cell viability assays.

3000 (Hs578T) or 10,000 (MDA-MB-231, BT20, BT549, MDA-MB-468, HCC1954, and MCF7) cells were seeded into 24-well plates. The next day, cells were treated with DMSO, doxorubicin (1–2 nM, as indicated in figures), and PARPi (300 nM) alone or in combination (doxorubicin/PARPi) every 2 days. For ROS scavenger studies, mitoTEMPO (10 μM) or NAC (5 mM), were added at the time of cell seeding. The following day, cells were treated with media containing doxorubicin and PARPi alone or in combination as described above. The scavengers were replenished every day. Cell counts were performed during the third and fifth day of treatment. Cell viability was evaluated by Trypan blue (Wisent Bio Products, catalog 609-130-EL) exclusion.

### Soft agar assay.

1.5 × 10^4^ Hs578T cells were plated into 1.5 mL of 20% FBS DMEM 0.4% Agar (Bioshop, catalog AGR001.500) over a layer of 2 mL 20% FBS DMEM 0.6% Agar in 6-well plates. The following day, cells were treated with DMSO, 1 nM doxorubicin, 300 nM PARPi, or a combination. The concentration of drugs was calculated considering the agar volume (3.5 mL) and then media was added (200 μL). Cells were treated every 3 days and monitored over a 10-day period. Images of 4 fields per well were acquired with an inverted light microscope using Infinity Capture software and analyzed using ImageJ software (NIH).

### Mammary fat pad injections.

1 × 10^6^ Hs578T cells, expressing VC or p66ShcA, were injected into the fourth mammary fat pad of 10- to 12-week-old SCID-Beige female mice. Upon first palpation, tumor growth was monitored using caliper measurements as described previously (78). When tumors reached 150 mm^3^, mice were randomized into 4 treatment groups: doxorubicin alone, PARPi alone, doxorubicin/PARPi in combination, and vehicle control. Doxorubicin (2.5 mg/Kg) was administered by i.p. injection every 3 days; PARPi (25 mg/Kg) and vehicle were administered via gavage daily. Throughout the course of treatment, tumor volumes were monitored every 2 days until the vehicle control group reached an approximate volume of 750 mm^3^. Breast tumors were fixed in 10% buffered formalin, or flash frozen in liquid nitrogen.

### Flow cytometry.

For cell cycle and cyt c release analysis, cells were cultured at 15,000 cells/cm^2^ and treated with DMSO, doxorubicin (10 nM), and PARPi (300 nM) alone or in combination for 48 hours. For cell cycle analysis, cells were trypsinized and washed with 3%FBS/PBS then fixed in 75% ethanol/PBS. Cells were then collected by centrifugation and washed with 3%FBS/PBS followed by PI (MilliporeSigma, catalog P4170) staining (25 μg/mL PI, 500 μg/mL RNAse, 3.6 mM sodium citrate) overnight at 4°C. The following day, cells were resuspended in 300 μL fresh 25 μg/mL PI. To examine cyt c release, its retention within the mitochondria was determined as previously described (79). After treatment, cells were trypsinized and counted. 500,000 cells were Live/Dead stained (Thermo Fisher, catalog L34957) for 15 minutes at room temperature, followed by a 45-minute incubation in 0.002% digitonin (MilliporeSigma, catalog D141). At this point, the positive control for cyt c release was also incubated in 25 μM alamethicin (Cayman Chemical, catalog 11425). After the detergent incubation, cells were fixed in 1% formaldehyde for 10 minutes. Formaldehyde was neutralized with Tris-Glycine buffer pH 9.1. Conjugated cyt c antibody diluted to 1:600 in staining buffer (BioLegend, catalog 612310) was added to the samples overnight at 4°C.

For apoptosis analysis, cells were cultured at 1500 cells/cm^2^ and treated with DMSO, doxorubicin (1 nM), and PARPi (300 nM) alone or in combination. Seventy-two hours later, 100,000 cells were collected and stained with Alexa Fluor 647 Annexin V (BioLegend, catalog 640912) according to the manufacturer’s protocol.

Cell cycle, cyt c release, and apoptosis were assessed through flow cytometry using the BD LSR Fortessa cell analyzer. Data analysis was performed with FlowJo software.

### Immunofluorescence.

Immunofluorescence was performed as previously described (80). 3000 (Hs578t) or 10,000 (MDA-MB-231) cells were seeded onto coverslips. The next day, cells were treated with DMSO, doxorubicin (1 nM), and PARPi (300 nM) alone or in combination for 48 hours. Cells were fixed in freshly prepared 2% paraformaldehyde (MilliporeSigma, catalog P6148) for 15 minutes. Cells were then incubated for 10 minutes in 0.3% Triton X-100, 1% BSA (Bioshop, catalog ALB001.500), and 2% normal goat serum (NGS; Wisent Bio Products, catalog 053-110) in PBS. Fixed and permeabilized cells were then blocked over 30 minutes in 3% BSA/ 2% NGS/PBS at room temperature. Cells were then incubated with anti-γH2AX primary antibody (MilliporeSigma, catalog JBW301, 1:500) in 1% BSA/PBS, for 1 hour at room temperature. Thereafter, cells were washed 3 times with 1%BSA/PBS and incubated with 1:2000 Mouse Alexa 488 nm (Invitrogen, catalog A11029) in 1%BSA/PBS for 1 hour at room temperature. Cells were washed as described above, stained with DAPI (1 μg/ml), and subjected to a final wash step. The coverslips were mounted onto glass slides using ProLong gold antifade reagent (Thermo Fisher, catalog P36930). Images were acquired using a Leica Widefield DM LB2 microscope and analyzed using ImageJ (NIH).

### Immunoblot analysis.

Cells were lysed with a buffer (20 mM Tris pH 7.5, 420 mM NaCl, 2 mM MgCl_2_, 1 mM EDTA, 10% glycerol, 0.5% NP40, 0.5% Triton X-100) supplemented with 5 mM NaF, protease and phosphatase inhibitor cocktail (PIN; 1 μg/ml chymostatin, catalog CHY222.10; 2 μg/ml antipain, catalog ANT604.10; 2 μg/ml leupeptin, catalog LEU001.10; 1 μg/ml Pepstatin, catalog PEP605.10; 2 μg/ml Aprotinin, catalog APR600.10; all from BioShop), and 5 mM NaVO_4_. Two volumes of lysis buffer (relative to cell pellet) were added to the collected cells and left on ice for 25 minutes. Lysates were centrifuged at 16,000*g*, 4°C, for 20 minutes. Protein concentration was measured by Pierce BCA Protein assay (Thermo Fisher, catalog 23227). Proteins were separated by SDS-PAGE, transferred onto polyvinylidene difluoride membranes, blocked with 5% milk TBST (Tris base 20 mM, NaCl 137 mM, 0.05% Tween 20), and incubated in primary antibody (1:5000 ShcA, MilliporeSigma, catalog 06-203; 1:5000 α-Tubulin, MilliporeSigma, catalog T5168). Secondary IgG antibodies conjugated to horseradish peroxidase (1:10,000) and ECL (Thermo Fisher, catalog 32106) were used for protein detection. See complete unedited blots in the supplemental material.

### Immunoprecipitation.

High-density cell cultures (15,000 cells/cm^2^) were treated with 10 nM doxorubicin or 300 nM PARPi alone or in combination for 48 hours. Cells were collected and lysed as described above. Protein concentration was determined by Pierce BCA Assay. A total of 1 mg protein was diluted in PLCγ lysis buffer (cytoplasmic buffer) supplemented with PIN, NaF, and NaVO_4_ (up to 300 μL total volume). The diluted lysates were nutated with FLAG antibody (MilliporeSigma, catalog F3165; 1:250) for 3 hours at 4°C. Cell lysates were then mixed with 30 μl bed volume of a 1:1 ratio of protein G-Sepharose 4 Fast Flow (GE Healthcare, catalog 17-0618-02) to PLCγ lysis buffer overnight. Beads were washed with PLCγ lysis buffer 3 times by centrifugation at 3000*g* for 2 minutes at 4°C. Proteins were eluted with cytoplasmic lysis buffer (with DTT) by incubating them at 95°C for 5 minutes. Eluate was collected by centrifugation at 5000*g* for 2 minutes at room temperature. Supernatant was collected, and SDS-Page Loading buffer was added to the eluate. Immunoblot procedures were performed as described above with 1:1000 pS36-p66ShcA (Abcam, catalog ab54518) and 1:5000 ShcA (MilliporeSigma, catalog 06-203) antibodies.

### Immunohistochemistry.

IHC analyses were performed on paraffin-embedded breast tumor samples, sectioned at 5 μm. Antigen retrieval was performed in sodium citrate (2.94 g Tris-sodium citrate [dihydrate] in 100 mL distilled water with 0.05% Tween20, pH was adjusted to 6.0 with 1 N HCl). Tumor sections were permeabilized with two 5-minute washes with TBST (0.05% Tween 20 in 1 mM Tris, pH 8, 15 mM NaCl). Slides were incubated with unconjugated avidin (BioLegend, catalog 927301) for 10 minutes at room temperature, followed by 5-minute TBST wash and 10-minute incubation with unconjugated biotin (BioLegend, catalog 927301). Slides were washed with TBST for 5 minutes and blocked with 10%BSA/TBS for 30 minutes. Breast tumor sections were incubated in primary antibody at 4°C overnight (1:500 KI-67, Abcam, catalog ab15580; 1:250 Cleaved Caspase-3, Cell Signaling Technologies, catalog 9661; 1:250 phospho-AMPK, Cell Signaling Technologies, catalog 2531; 1:500 phospho-ACC, Cell Signaling Technologies, catalog 3661). Following three 5-minutes washes with TBST, breast tumor sections were incubated in avidin/biotinylated complex (Vectastain, Vector Laboratories, catalog VECTPK4000) for 30 minutes, followed by three 5-minute washes. Finally, the staining was developed using diaminobenzidine substrate (Vector Laboratories, catalog SK-4105). Tissues were counterstained with 20% hematoxylin (Fisher Scientific, catalog SH26). Staining with monoclonal antibodies (1:2000 8-oxodG, Trevigen, catalog 4354-MC and 1:500 γH2AX, MilliporeSigma, catalog JBW301) was performed with the Mouse on Mouse Polymer IHC Kit (Abcam, catalog ab127055) according to the manufacturer’s instructions. Slides were scanned using a ScanScope XT Digital Slide Scanner (Aperio), and data were analyzed using Image Scope software.

### Seahorse respirometry.

OCRs were measured using an XF96 Seahorse instrument (Extracellular Flux Analyzer, Agilent) per the manufacturer’s instructions. Briefly, cells were seeded at 3000 cells per well in 80 μL 10%FBS DMEM media. After attachment (1 hour) cells were treated with DMSO, 10 nM doxorubicin, or 300 nM PARPi alone or in combination for 36 hours in a 37°C incubator. Cells were then washed twice with XF base media (Agilent), supplemented with 25 mmol/L glucose, 4 mmol/L glutamine, and 1 mmol/L sodium pyruvate. A final volume of 180 μL supplemented XF media was added, and the plate was set to incubate for 1 hour in a CO_2_-free incubator at 37°C. OCR was obtained by repeated cycles of mix (3 minutes), pause (3 minutes), and measurement (3 minutes). Measurements were normalized on protein content at the end of the experiment.

### In vivo drug sensitivity.

In vivo drug sensitivity studies were done in NOD.Cg-*Prkdc^scid^ Il2rg^tm1Wjl^*/SzJ (NSG) mice (The Jackson Laboratory) using a 1 × 1 × 1 approach (81). Briefly, tumor fragments orthotopically engrafted in mice were allowed to grow to 100 mm^3^ before initiating a 28-day treatment regimen. The following drug regimens were used: 3 mg/kg doxorubicin (in 0.9% normal saline) i.v. weekly, 4 mg/kg cisplatin (in 0.9% normal saline) i.v. weekly. Tumor dimensions were measured twice per week; volume was calculated according to the formula *V* = (length × width^2^)/2. BestAvgResponse was calculated as previously described (81). Briefly, response was determined by comparing tumor volume change at time *t* to its baseline with the formula Δ*V_t_* = ([*V_t_* – *V_initial_*]/*V_initial_*) × 100. The BestAvgResponse was calculated as the minimum of the average of Δ*V_t_* from *t* = 0 to *t* for *t* ≥14 days.

### Statistics.

The statistical analysis and graphing functions were performed using Prism Graphpad 7 software. Data from viability assays, cell cycle staining, annexin V staining, cyt c release, and Seahorse respirometry were analyzed with a 2-way ANOVA (Tukey’s multiple comparisons test). Soft agar, γH2AX immunofluorescence, pS36-p66ShcA densitometry, and IHC positivity (cleaved caspase-3, KI-67, γH2AX, pACC, ACC, pAMPK, AMPK, and 8-oxoG) were analyzed with 1-way ANOVA (Tukey’s multiple comparisons test). EOB drug curves were analyzed using paired, 2-tailed *t* tests, and IC_50_ concentrations were calculated with a 4-parameter nonlinear regression. Finally, a mixed-effect analysis and Sidak’s multiple comparison tests were performed for tumor growth curves. A *P* value of less than 0.05 was considered significant.

### Study approval.

All animal studies were approved by the Animal Resources Council at McGill University and comply with guidelines set by the Canadian Council of Animal Care.

## Author contributions

ECC, ST, RA, GD, DM, and JH performed the experiments. ECC, MW, and JUS analyzed the data. CA and MP provided reagents and expertise. PS generated reagents that were essential for this study. ECC, MW, and JUS wrote and edited the manuscript. MW and JUS designed the experiments and supervised the study.

## Supplementary Material

Supplemental data

## Version 1 01/20/2021

In-Press Preview

## Version 2 01/21/2021

In-Press Preview

## Version 3 02/22/2021

Electronic publication

## References

[1] Dent R (2007). Triple-negative breast cancer: clinical features and patterns of recurrence. Clin Cancer Res.

[2] Penault-Llorca F, Viale G (2012). Pathological and molecular diagnosis of triple-negative breast cancer: a clinical perspective. Ann Oncol.

[3] Oakman C (2010). Management of triple negative breast cancer. Breast.

[4] Goldhirsch A (2009). Thresholds for therapies: highlights of the St Gallen international expert consensus on the primary therapy of early breast cancer 2009. Ann Oncol.

[5] Anders CK, Carey LA (2009). Biology, metastatic patterns, and treatment of patients with triple-negative breast cancer. Clin Breast Cancer.

[6] Li X (2017). Triple-negative breast cancer has worse overall survival and cause-specific survival than non-triple-negative breast cancer. Breast Cancer Res Treat.

[7] Pareja F (2016). Triple-negative breast cancer: the importance of molecular and histologic subtyping, and recognition of low-grade variants. NPJ Breast Cancer.

[8] Kandoth C (2013). Mutational landscape and significance across 12 major cancer types. Nature.

[9] Synnott N (2017). Mutant p53: a novel target for the treatment of patients with triple-negative breast cancer?. Int J Cancer.

[10] Shah SP (2012). The clonal and mutational evolution spectrum of primary triple negative breast cancers. Nature.

[11] Lips EH (2013). Triple-negative breast cancer: BRCAness and concordance of clinical features with BRCA1-mutation carriers. Br J Cancer.

[12] Cancer Genome Atlas Network (2012). Comprehensive molecular portraits of human breast tumors. Nature.

[13] Stephens PJ (2012). The landscape of cancer genes and mutational processes in breast cancer. Nature.

[14] Frederick AM (2012). Sequence analysis of mutations and translocations across breast cancer subtypes. Nature.

[15] Ashworth A, Lord CJ (2016). BRCAness revisited. Nat Rev Cancer.

[16] Herceg Z, Wang Z-Q (2001). Functions of poly(ADP-ribose) polymerase (PARP) in DNA repair, genomic integrity and cell death. Mutat Res.

[17] Altmeyer M (2009). Molecular mechanism of poly(ADP-ribosyl)ation by PARP1 and identification of lysine residues as ADP-ribose acceptor sites. Nucleic Acids Res.

[18] Marques M (2019). Oncogenic activity of poly (ADP-ribose) glycohydrolase. Oncogene.

[19] Chiou S-H (2013). Poly(ADP-ribose) polymerase 1 regulates nuclear reprogramming and promotes iPSC generation without c-Myc. J Exp Med.

[20] Liu C (2017). The role of poly ADP-ribosylation in the first wave of DNA damage response. Nucleic Acids Res.

[21] Farmer H (2005). Targeting the DNA repair defect in *BRCA* mutant cells as a therapeutic strategy. Nature.

[22] Bryant HE (2005). Specific killing of BRCA2-deficient tumours with inhibitors of poly(ADP-ribose) polymerase. Nature.

[23] Zimmermann M (2018). CRISPR screens identify genomic ribonucleotides as a source of PARP-trapping lesions. Nature.

[24] De Lorenzo SB (2013). The elephant and the blind men: making sense of PARP inhibitors in homologous recombination deficient tumor cells. Front Oncol.

[25] Pascal JM (2018). The comings and goings of PARP-1 in response to DNA damage. DNA Repair (Amst).

[26] Pulliam N (2018). An effective epigenetic-PARP inhibitor combination therapy for breast and ovarian cancers independent of BRCA mutations. Clin Cancer Res.

[27] Hou D (2018). Increased oxidative stress mediates the antitumor effect of PARP inhibition in ovarian cancer. Redox Biol.

[28] Liu Q (2018). PARP-1 inhibition with or without ionizing radiation confers reactive oxygen species-mediated cytotoxicity preferentially to cancer cells with mutant TP53. Oncogene.

[29] Yin Z-X (2017). PARP-1 inhibitors sensitize HNSCC cells to APR-246 by inactivation of thioredoxin reductase 1 (TrxR1) and promotion of ROS accumulation. Oncotarget.

[30] Migliaccio E (1999). The p66^shc^ adaptor protein controls oxidative stress response and life span in mammals. Nature.

[31] Giorgio M (2005). Electron transfer between cytochrome c and p66Shc generates reactive oxygen species that trigger mitochondrial apoptosis. Cell.

[32] Ventura A (2002). The p66Shc longevity gene is silenced through epigenetic modifications of an alternative promoter. J Biol Chem.

[33] Migliaccio E (1997). Opposite effects of the p52shc/p46shc and p66shc splicing isoforms on the EGF receptor-MAP kinase-fos signalling pathway. EMBO J.

[34] Ursini-Siegel J, Muller WJ (2008). The ShcA adaptor protein is a critical regulator of breast cancer progression. Cell Cycle.

[35] Hudson J (2014). p66ShcA promotes breast cancer plasticity by inducing an epithelial-to-mesenchymal transition. Mol Cell Biol.

[36] Pacini S (2004). p66SHC promotes apoptosis and antagonizes mitogenic signaling in T cells. Mol Cell Biol.

[37] Yang CP, Horwitz SB (2000). Taxol mediates serine phosphorylation of the 66-kDa Shc isoform. Cancer Res.

[38] Pinton P (2007). Protein kinase C beta and prolyl isomerase 1 regulate mitochondrial effects of the life-span determinant p66Shc. Science.

[39] Galimov ER (2010). The role of p66shc in oxidative stress and apoptosis. Acta Naturae.

[40] Zhen Y (1999). Definition of the interaction domain for cytochrome c on cytochrome c oxidase. I. Biochemical, spectral, and kinetic characterization of surface mutants in subunit ii of Rhodobacter sphaeroides cytochrome aa(3). J Biol Chem.

[41] Orsini F (2004). The life span determinant p66Shc localizes to mitochondria where it associates with mitochondrial heat shock protein 70 and regulates trans-membrane potential. J Biol Chem.

[42] Alli E (2009). Defective repair of oxidative DNA damage in triple-negative breast cancer confers sensitivity to inhibition of poly(ADP-ribose) polymerase. Cancer Res.

[43] Wang H (2020). Synergistic lethality between PARP-trapping and alantolactone-induced oxidative DNA damage in homologous recombination-proficient cancer cells. Oncogene.

[44] Doroshow JH (1986). Role of hydrogen peroxide and hydroxyl radical formation in the killing of Ehrlich tumor cells by anticancer quinones. Proc Natl Acad Sci U S A.

[45] Dasari S, Bernard Tchounwou P (2014). Cisplatin in cancer therapy: molecular mechanisms of action. Eur J Pharmacol.

[46] Thorn CF (2011). Doxorubicin pathways: pharmacodynamics and adverse effects. Pharmacogenet Genomics.

[47] Jones P (2009). Discovery of 2-{4-[(3S)-Piperidin-3-yl]phenyl}-2H-indazole-7-carboxamide (MK-4827): a novel oral poly(ADP-ribose)polymerase (PARP) inhibitor efficacious in BRCA-1 and -2 mutant tumors. J Med Chem.

[48] Rodler ET (2016). Phase I study of veliparib (ABT-888) Combined with cisplatin and vinorelbine in advanced triple-negative breast cancer and/or BRCA mutation-associated breast cancer. Clin Cancer Res.

[49] Sharma P (2020). Results of a phase II randomized trial of cisplatin +/- veliparib in metastatic triple-negative breast cancer (TNBC) and/or germline BRCA-associated breast cancer (SWOG S1416). J Clin Oncol.

[50] Jelinic P, Levine DA (2014). New insights into PARP inhibitors’ effect on cell cycle and homology-directed DNA damage repair. Mol Cancer Ther.

[51] Clark CC (2012). Enhancement of synthetic lethality via combinations of ABT-888, a PARP inhibitor, and carboplatin in vitro and in vivo using BRCA1 and BRCA2 isogenic models. Mol Cancer Ther.

[52] Hastak K (2010). Synergistic chemosensitivity of triple-negative breast cancer cell lines to poly(ADP-Ribose) polymerase inhibition, gemcitabine, and cisplatin. Cancer Res.

[53] Kuo LJ, Yang L-X (2008). Gamma-H2AX - a novel biomarker for DNA double-strand breaks. In Vivo.

[54] Soliman MA (2014). The adaptor protein p66Shc inhibits mTOR-dependent anabolic metabolism. Sci Signal.

[55] Brown KK (2017). Adaptive reprogramming of *de novo* pyrimidine synthesis is a metabolic vulnerability in triple-negative breast cancer. Cancer Discov.

[56] Tao Z (2006). Inhibition of cellular respiration by doxorubicin. Chem Res Toxicol.

[57] Murata MM (2019). NAD+ consumption by PARP1 in response to DNA damage triggers metabolic shift critical for damaged cell survival. Mol Biol Cell.

[58] Lewis K (2020). p66ShcA functions as a contextual promoter of breast cancer metastasis. Breast Cancer Res.

[59] Gelmon KA (2011). Olaparib in patients with recurrent high-grade serous or poorly differentiated ovarian carcinoma or triple-negative breast cancer: a phase 2, multicentre, open-label, non-randomised study. Lancet Oncol.

[60] Fong PC (2009). Inhibition of poly(ADP-ribose) polymerase in tumors from BRCA mutation carriers. N Engl J Med.

[61] Robson M (2017). Olaparib for metastatic breast cancer in patients with a germline BRCA mutation. N Engl J Med.

[62] Livraghi L, Garber JE (2015). PARP inhibitors in the management of breast cancer: current data and future prospects. BMC Med.

[63] Loibl S (2018). Addition of the PARP inhibitor veliparib plus carboplatin or carboplatin alone to standard neoadjuvant chemotherapy in triple-negative breast cancer (BrighTNess): a randomised, phase 3 trial. Lancet Oncol.

[64] Marques M (2015). Chemotherapy reduces PARP1 in cancers of the ovary: implications for future clinical trials involving PARP inhibitors. BMC Med.

[65] Makvandi M (2018). A PET imaging agent for evaluating PARP-1 expression in ovarian cancer. J Clin Invest.

[66] Jackson JG (2000). Elevated levels of p66 Shc are found in breast cancer cell lines and primary tumors with high metastatic potential. Clin Cancer Res.

[67] Liedtke C (2008). Response to neoadjuvant therapy and long-term survival in patients with triple-negative breast cancer. J Clin Oncol.

[68] Isakoff SJ (2010). Triple-negative breast cancer: role of specific chemotherapy agents. Cancer J.

[69] Abbotts R (2019). DNA methyltransferase inhibitors induce a BRCAness phenotype that sensitizes NSCLC to PARP inhibitor and ionizing radiation. Proc Natl Acad Sci U S A.

[70] Muvarak NE (2016). Enhancing the cytotoxic effects of PARP inhibitors with DNA demethylating agents - a potential therapy for cancer. Cancer Cell.

[71] Korkmaz-Icöz S (2018). Olaparib protects cardiomyocytes against oxidative stress and improves graft contractility during the early phase after heart transplantation in rats: Protection by olaparib against cardiac injury. Br J Pharmacol.

[72] Muñoz-Gámez JA (2005). PARP inhibition sensitizes p53-deficient breast cancer cells to doxorubicin-induced apoptosis. Biochem J.

[73] Ott M (2002). Cytochrome c release from mitochondria proceeds by a two-step process. Proc Natl Acad Sci U S A.

[74] Shidoji Y (1999). Loss of molecular interaction between cytochrome c and cardiolipin due to lipid peroxidation. Biochem Biophys Res Commun.

[75] Kagan VE (2005). Cytochrome c acts as a cardiolipin oxygenase required for release of proapoptotic factors. Nat Chem Biol.

[76] Muñoz-Gámez JA (2011). Inhibition of poly (ADP-ribose) polymerase-1 enhances doxorubicin activity against liver cancer cells. Cancer Lett.

[77] Magan N (2012). Treatment with the PARP-inhibitor PJ34 causes enhanced doxorubicin-mediated cell death in HeLa cells. Anticancer Drugs.

[78] Ahn R (2013). The ShcA PTB domain functions as a biological sensor of phosphotyrosine signaling during breast cancer progression. Cancer Res.

[79] Ryan J (2016). iBH3: simple, fixable BH3 profiling to determine apoptotic priming in primary tissue by flow cytometry. Biol Chem.

[80] Hilmi K (2017). CTCF facilitates DNA double-strand break repair by enhancing homologous recombination repair. Sci Adv.

[81] Gao H (2015). High-throughput screening using patient-derived tumor xenografts to predict clinical trial drug response. Nat Med.

