# Supplementary material for: p66ShcA potentiates the cytotoxic response of triple-negative breast cancers to PARP inhibitors
Source: JCI Insight. 2021 Feb 22;6(4):e138382. doi: 10.1172/jci.insight.138382 (PMC7934920; doi:10.1172/jci.insight.138382)

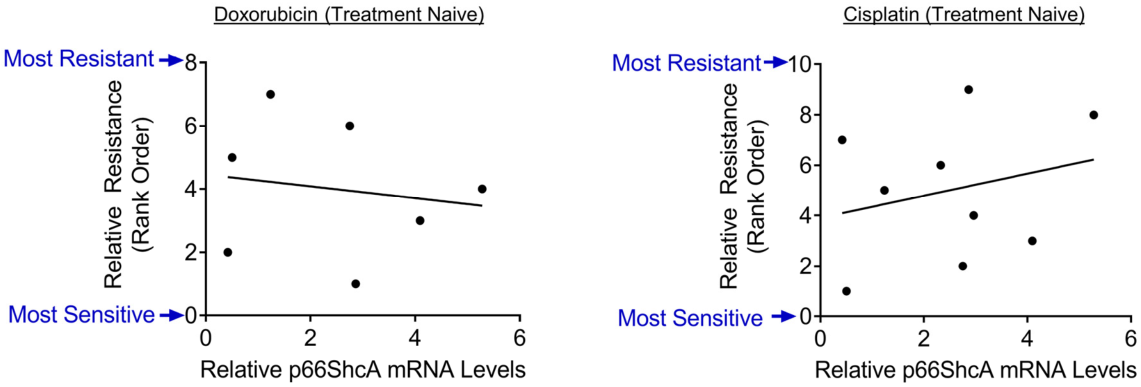

Supplemental Figure 1: Correlation between endogenous p66ShcA RNA transcript (ENST00000368445.9) levels and Best Average Response (ordered-ranked) obtained from chemogenomic profiling of TNBC PDXs in NOD.Cg-*Prkdc*^{scid} *Il2rg*^{tm1Wjl}/SzJ (NSG) mice exposed to 3 mg/kg doxorubicin (in 0.9% normal saline) intravenous (IV) weekly (left) or to 4 mg/kg cisplatin (in 0.9% normal saline) IV weekly (right). TNBC PDXs were established from chemo-naïve patients. Linear regression was generated based on a Spearman Correlation.

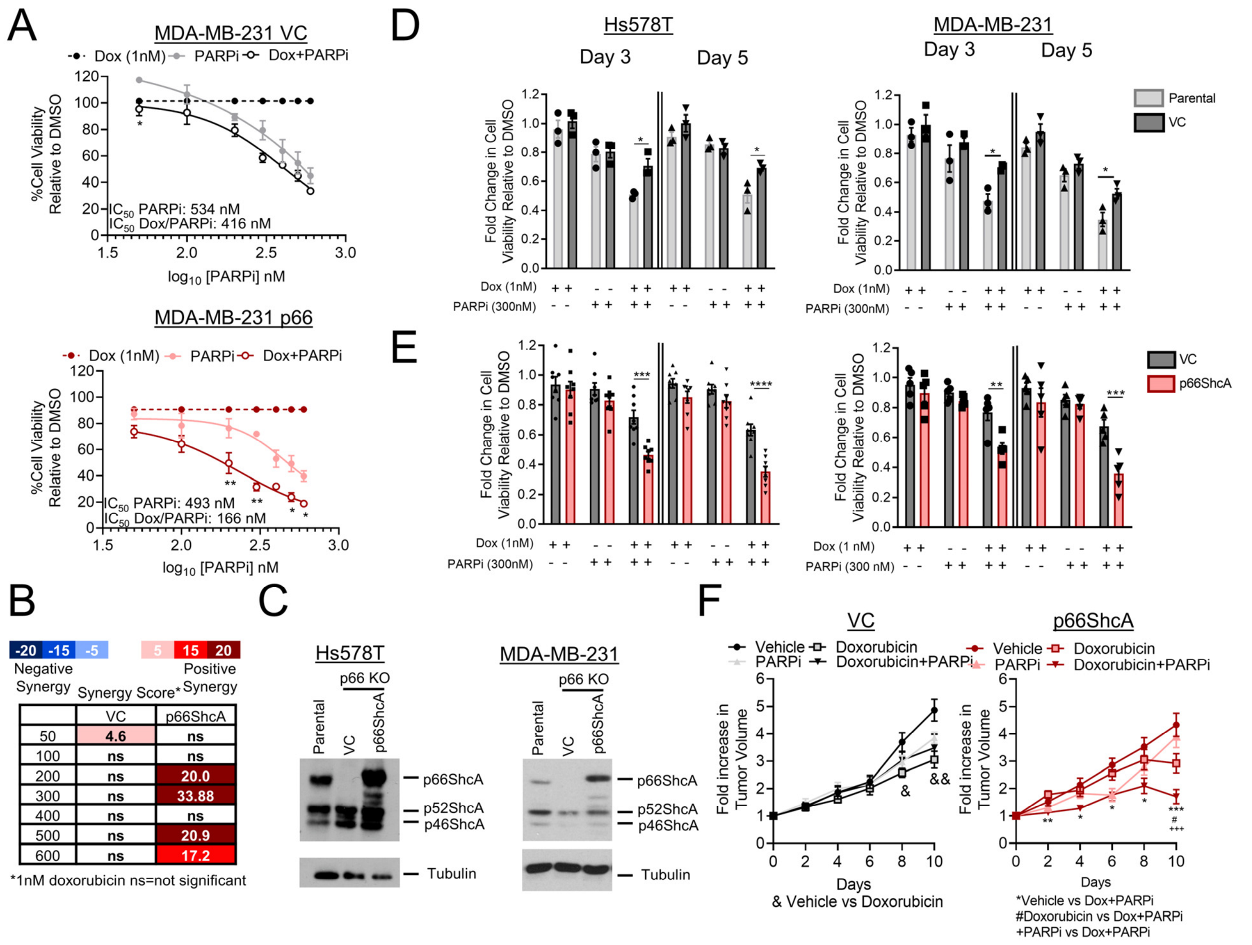

Supplemental Figure 2: (A) Cells were cultured in DMSO, doxorubicin (1 nM), PARPi (50-600 nM), alone or combined for 5 days. Viable cells were quantified by trypan blue exclusion. Data is shown as mean of means of fold change in viability relative to DMSO ± SEM (n=3 experiments). The PARPi IC_{50} values are shown, either as a monotherapy or in combination with doxorubicin. **(B)** Excess over bliss scores. **(C)** Relative p66ShcA levels in parental, p66ShcA-null (VC) or p66ShcA re-expressing cells **(D, E)** Cells were cultured as described and viability was quantified by trypan blue exclusion. Fold change in viability relative to DMSO ± SEM (n=3-7 biological replicates). **(F)** Same data as Figure 1E whereby VC- and p66ShcA-expressing tumors were plotted independently. **P*<0.05; ***P*<0.01; ****P*<0.001; *****P*<0.0001 by 2-tailed t-test **(A)**, two-way ANOVA/Tukey's multiple comparisons test **(D, E)**, mixed-effect analysis/Tukey's multiple comparisons test **(F)**.

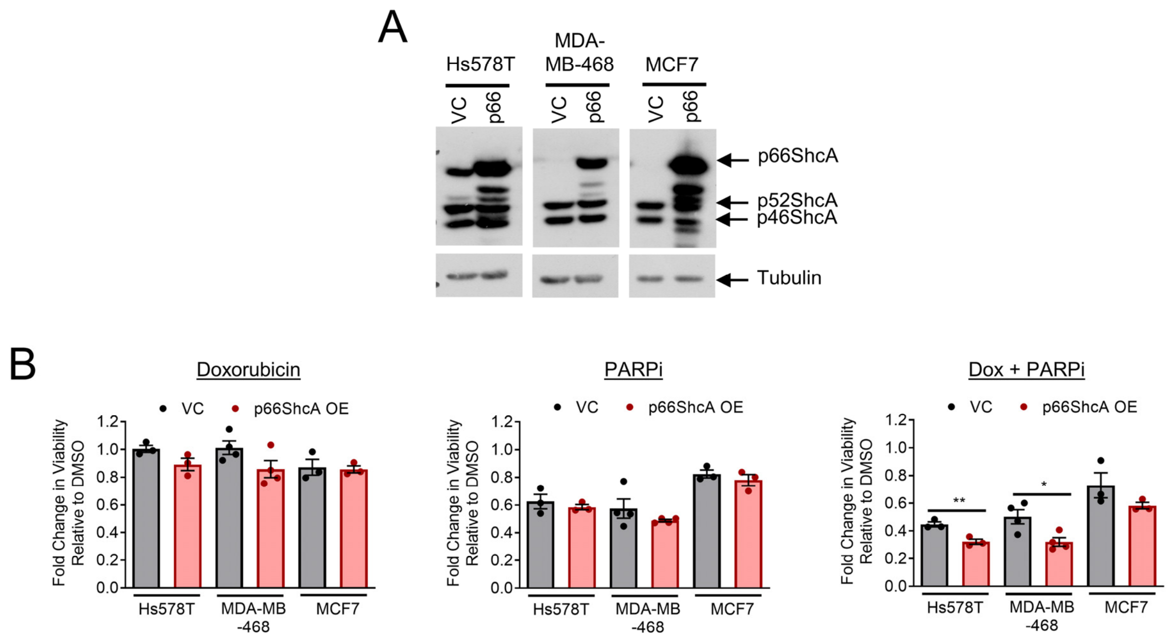

Supplemental Figure 3: Increased p66ShcA levels sensitizes TNBCs to doxorubicin in combination with PARP inhibitors. (A) ShcA and Tubulin immunoblot analysis of parental Hs578T, MDA-MB-468 and MCF7 cells expressing vector control plasmid (VC) or stably overexpressing p66ShcA (p66). **(B)** Cells were cultured in DMSO, doxorubicin and PARPi, alone or combined 5 days using the following concentrations: Hs578T: 2 nM doxorubicin/300 nM PARPi; MDA-MB-468 1 nM doxorubicin/100 nM PARPi; MCF7: 2 nM doxorubicin/300 nM PARPi. Viable cells were quantified by trypan blue exclusion. Data is shown as mean of means of fold change in the number of viable cells relative to DMSO ± SEM (n=3-4 independent experiments). *$P<0.05$;**$P<0.01$ by 2-tailed t-test.

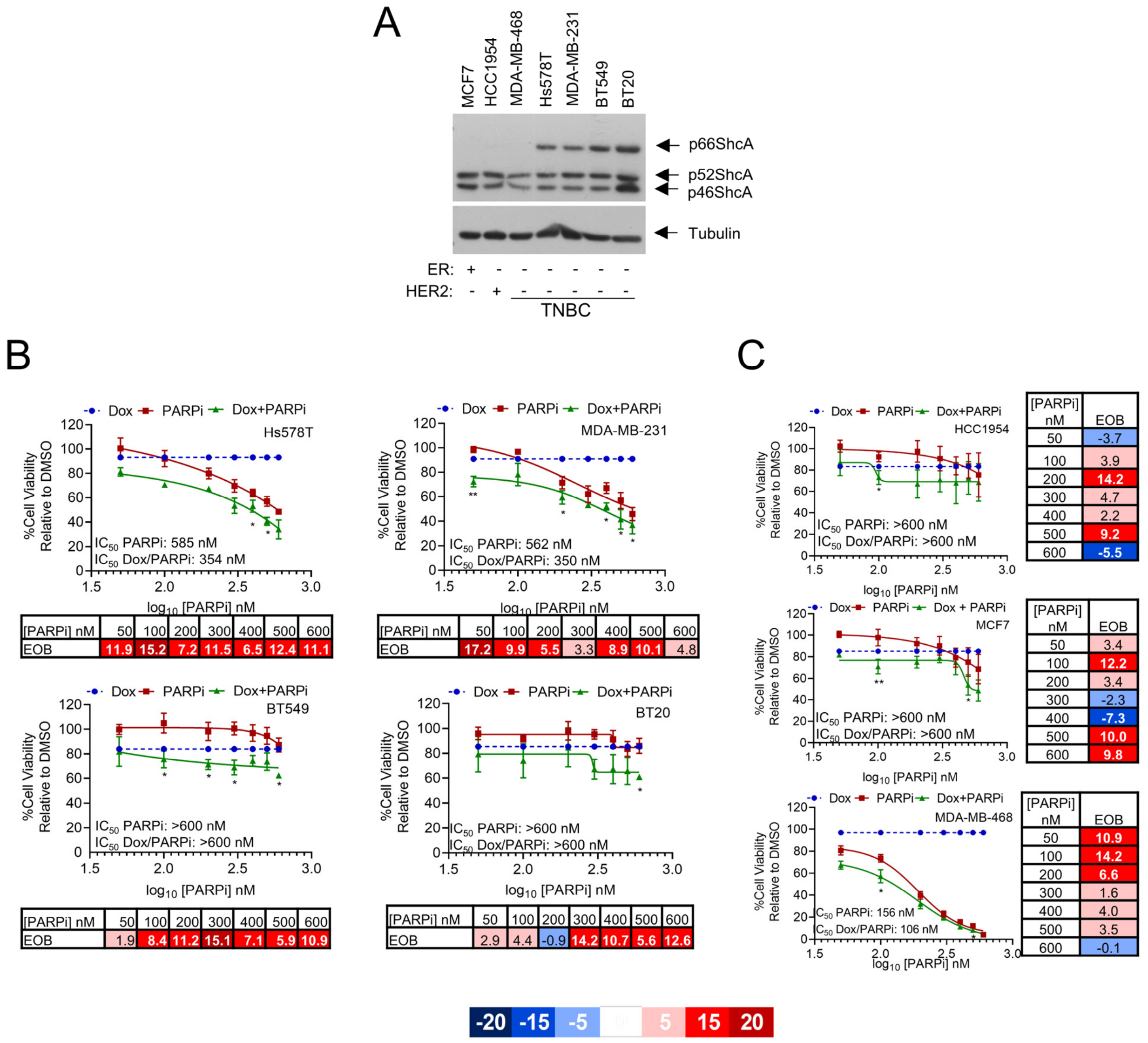

Supplemental Figure 4: Elevated p66ShcA levels are not associated with stronger synergy of breast cancer cells to doxorubicin/PARPi combination therapy. (A) ShcA and Tubulin immunoblot analysis of a panel of human breast cancer cell lines that differ based on their ER and/or HER2 status. Breast cancer cell lines that were either **(B)** p66ShcA positive or **(C)** lacked endogenous p66ShcA levels were cultured in DMSO, doxorubicin, PARPi (50-600 nM), alone or combined for 5 days. Doxorubicin was added to a final concentration of 1 nM (MDA-MB-468) or 2 nM (MCF7, HCC1954, Hs578T, MDA-MB-231, BT549, BT20). Viable cells were quantified by trypan blue exclusion. Data is shown as mean of means of fold change in the number of viable cells relative to DMSO ± SEM (n=3 independent experiments) Data for MDA-MB-468 is shown as mean of 2 independent experiments ± SD (n=6 technical repeats). **P*<0.05; ***P*<0.01 by 2-tailed t-test.

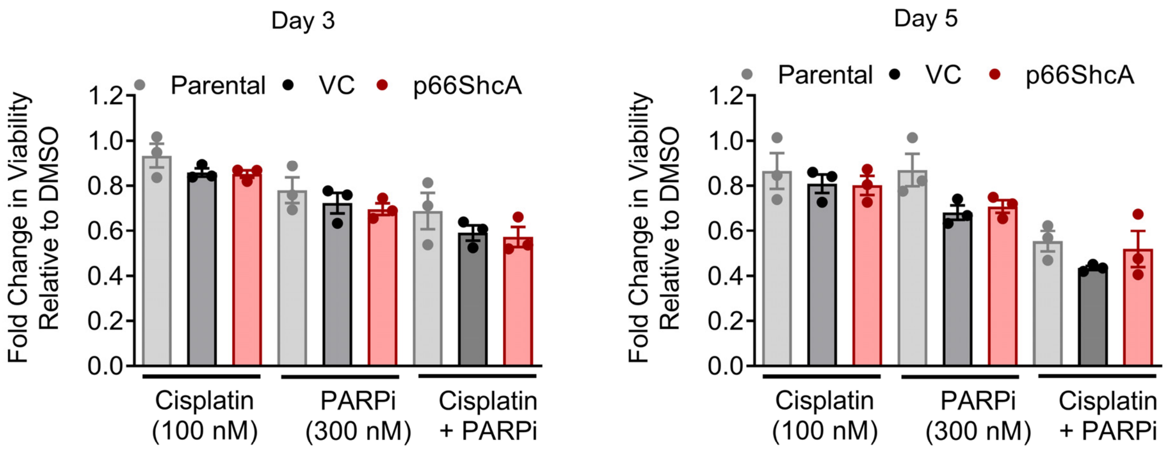

Supplemental Figure 5: p66ShcA does not impact the sensitivity of breast cancer cells to cisplatin in combination with PARP inhibitors. Hs578T cells were cultured in DMSO, cisplatin (100 nM), PARPi (300 nM), alone or combined for 3 or 5 days. Viable cells were quantified by trypan blue exclusion. Data is shown as mean of means of fold change in the number of viable cells relative to DMSO ± SEM (n=3 independent experiments).

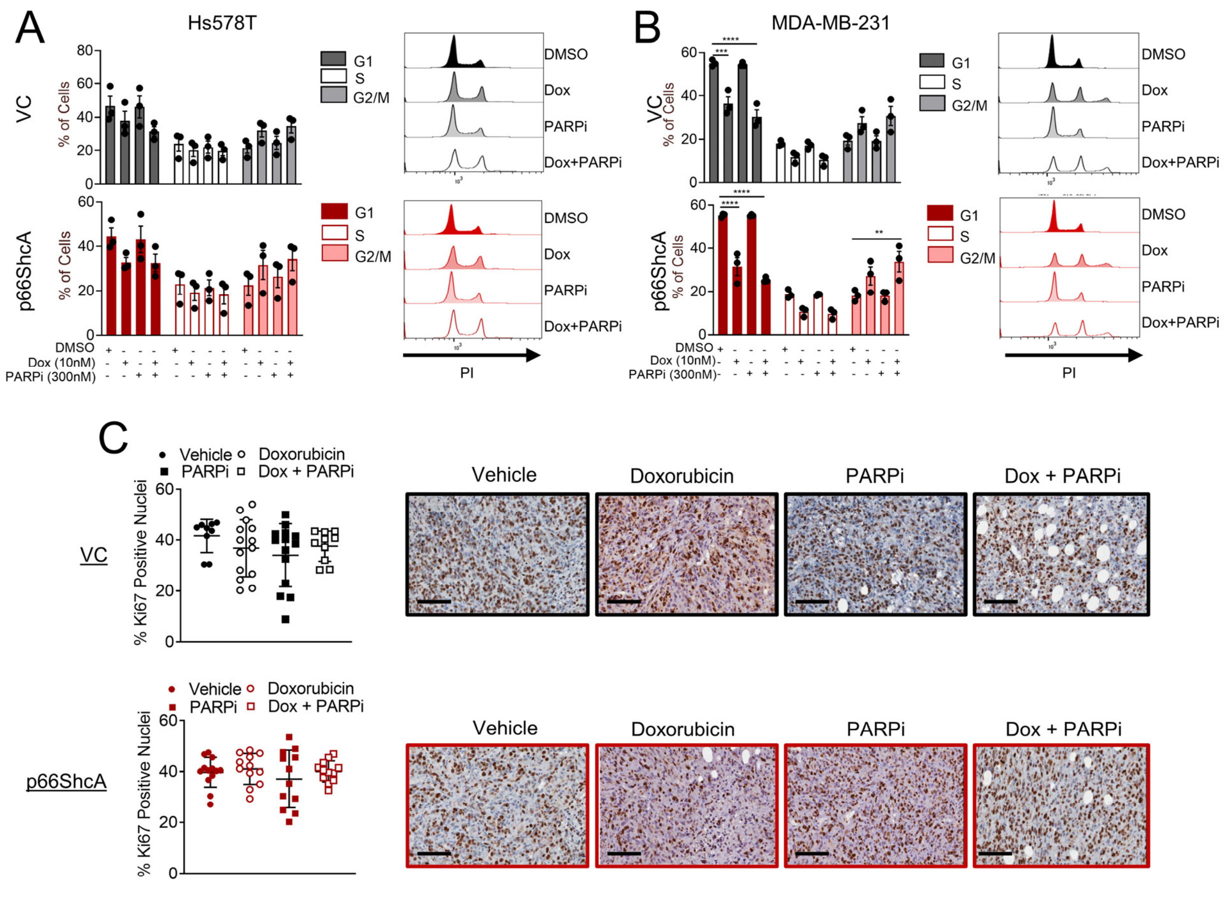

Supplemental Figure 6: VC or p66ShcA-expressing Hs578T **(A)** and MDA-MB-231 **(B)** cells were treated with doxorubicin (10 nM), PARPi (300 nM), alone or in combination. PI staining/flow cytometry was performed to measure DNA content and determine cell cycle distribution. The data is shown as average % of cells in G1, S or G2/M phase of the cell cycle ± SEM (n=3 independent experiments). Representative histograms of PI content are shown (Scale bars: 100μm). **(C)** The proliferative rate of VC and p66ShcA expressing Hs578T tumors (Figure 1E) was determined by Ki67 immunohistochemical staining. The data is depicted as % average of positive Ki-67 nuclei ± SEM (n=10-15 tumors per group) Representative images are shown. $^{**}P<0.01$; $^{***}P<0.001$; $^{****}P<0.0001$ by two-way ANOVA/ Tukey's multiple comparisons test **(A, B)**.

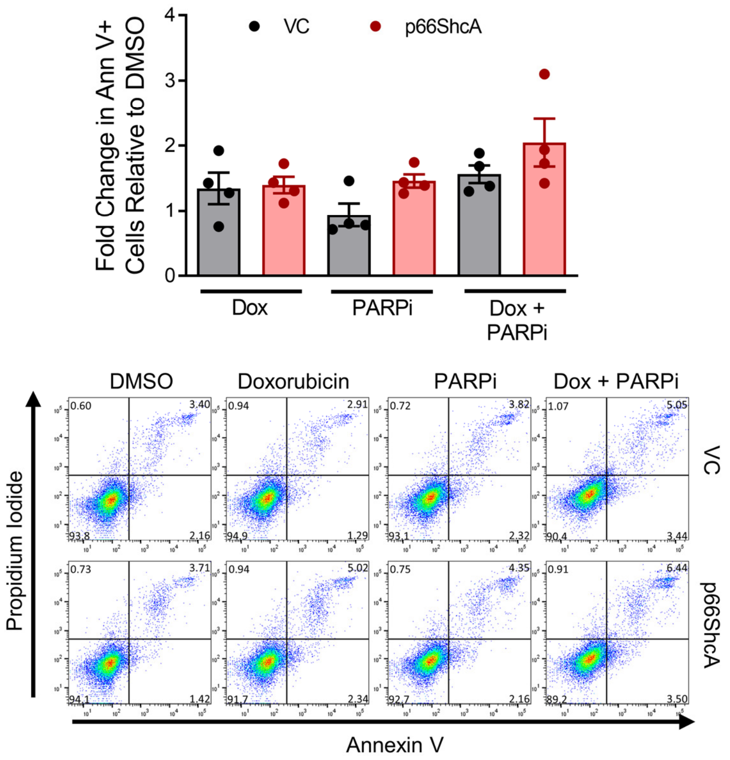

Supplemental Figure 7: p66ShcA does not impact apoptosis induced by doxorubicin/PARPi in MDA-MB-231 cells. p66ShcA-null (VC) or p66ShcA-reconstituted MDA-MB-231 cells were treated with DMSO, doxorubicin (1nM) and PARPi (300nM), alone or in combination for 72h and assessed by Annexin V staining. Results are presented as average “fold change” in % of Annexin V+ cells relative to DMSO ± SEM (n=4 independent experiments). Representative dot plots are shown.

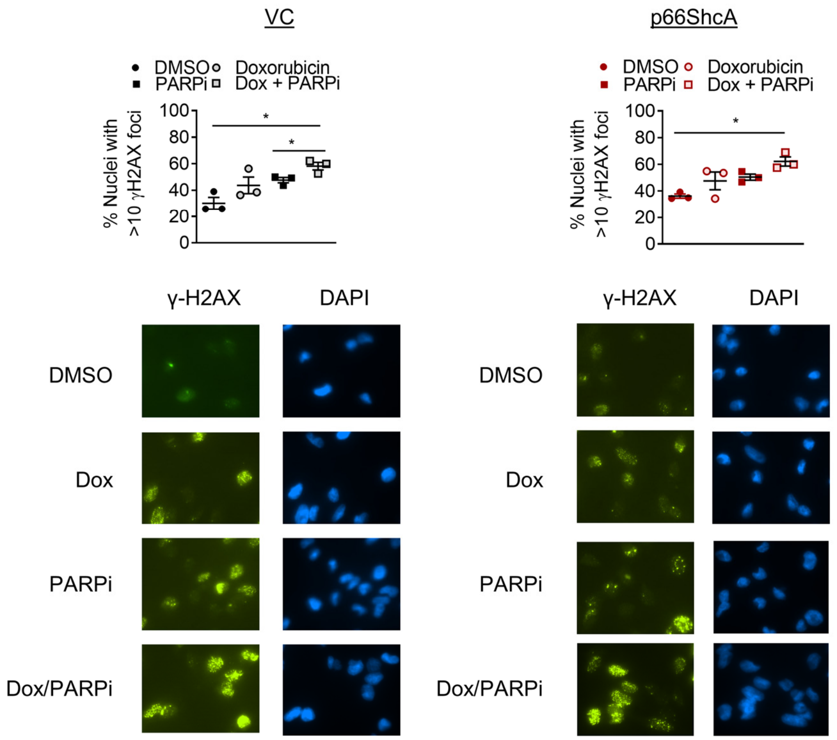

Supplemental Figure 8: The DNA damage response is unaffected by p66ShcA in response to doxorubicin/PARPi combination therapy in an independent TNBC model. VC or p66ShcA-expressing MDA-MB-231 cells were treated with doxorubicin (1nM) and PARPi (300nM), alone or in combination for 48h. Double-strand DNA breaks were assessed by γH2AX immunofluorescent staining. % nuclei with >10 γH2AX foci was quantified ± SEM (n=3 biological replicates). Representative images are shown. *P<0.05; **P<0.01 by one-way ANOVA/ Tukey's multiple comparisons test.

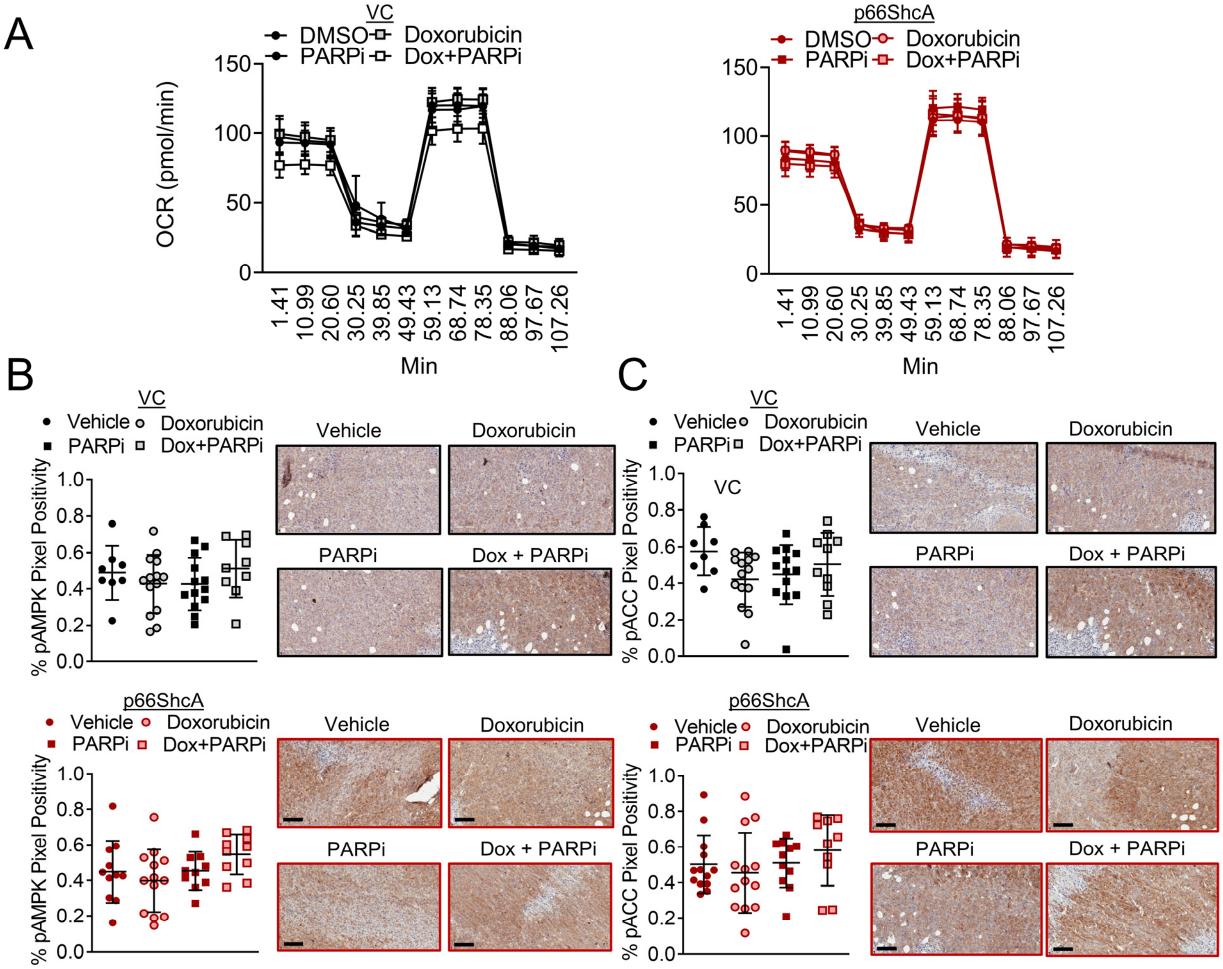

Supplemental Figure 9: (A) VC or p66ShcA-expressing Hs578T cells were treated with doxorubicin (10 nM), PARPi (300 nM), alone or in combination for 36 hours. OCR measurements were determined at the times indiciated. The graph is a representative experiment and shows average OCR ± SD (n=2 independent experiments with 7-8 technical repeats). **(B, C)** VC and p66ShcA expressing Hs578T tumors were treated with doxorubicin alone, PARPi alone, doxorubicin/PARPi combination or vehicle control (Fig 1E). Energetic stress was evaluated by **(B)** phospho-AMPK (Thr172) and **(C)** phospho-Acetyl-CoA Carboxylase (Ser79) immunohistochemical staining. The data is depicted as average % pixel positivity ± SEM (n=8-15 tumors per group). Representative images of the IHC staining illustrating pAMPK and pACC positivity are shown (Scale bars: 100μm).

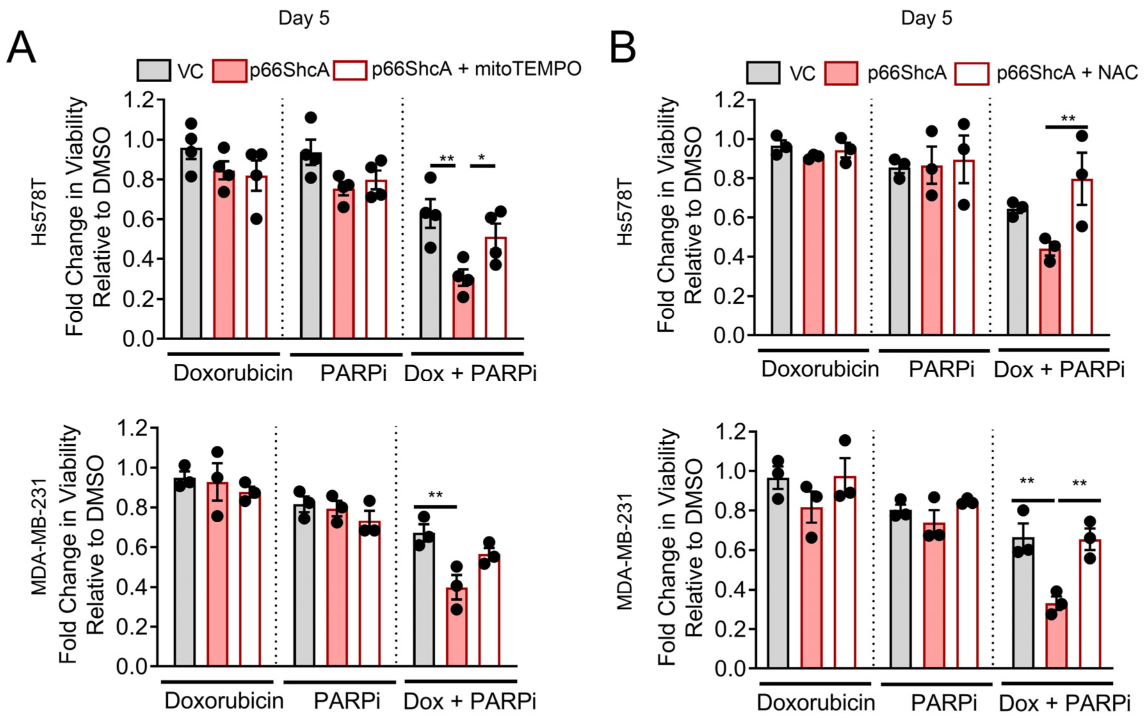

Supplemental Figure 10: VC and p66ShcA-expressing Hs578T and MDA-MB-231 cells were treated with doxorubicin (1nM) and PARPi (300 nM), alone or in combination in presence or absence of **(A)** MitoTEMPO (10μM) or **(B)** NAC (5mM) for 5 days. Cell viability was determined by trypan blue exclusion. Data is shown as mean of means of fold change in the number of viable cells relative to DMSO ± SEM (n=3-4 biological replicates) *P<0.05 **P < 0.01 by two-way ANOVA/ Tukey's multiple comparisons test.

Uncropped film used for Figure 4A

Top part of the film was used for Figure 4A IP FLAG Western Blot Ps36-p66ShcA. The + mark represents 75kDa of the protein ladder.

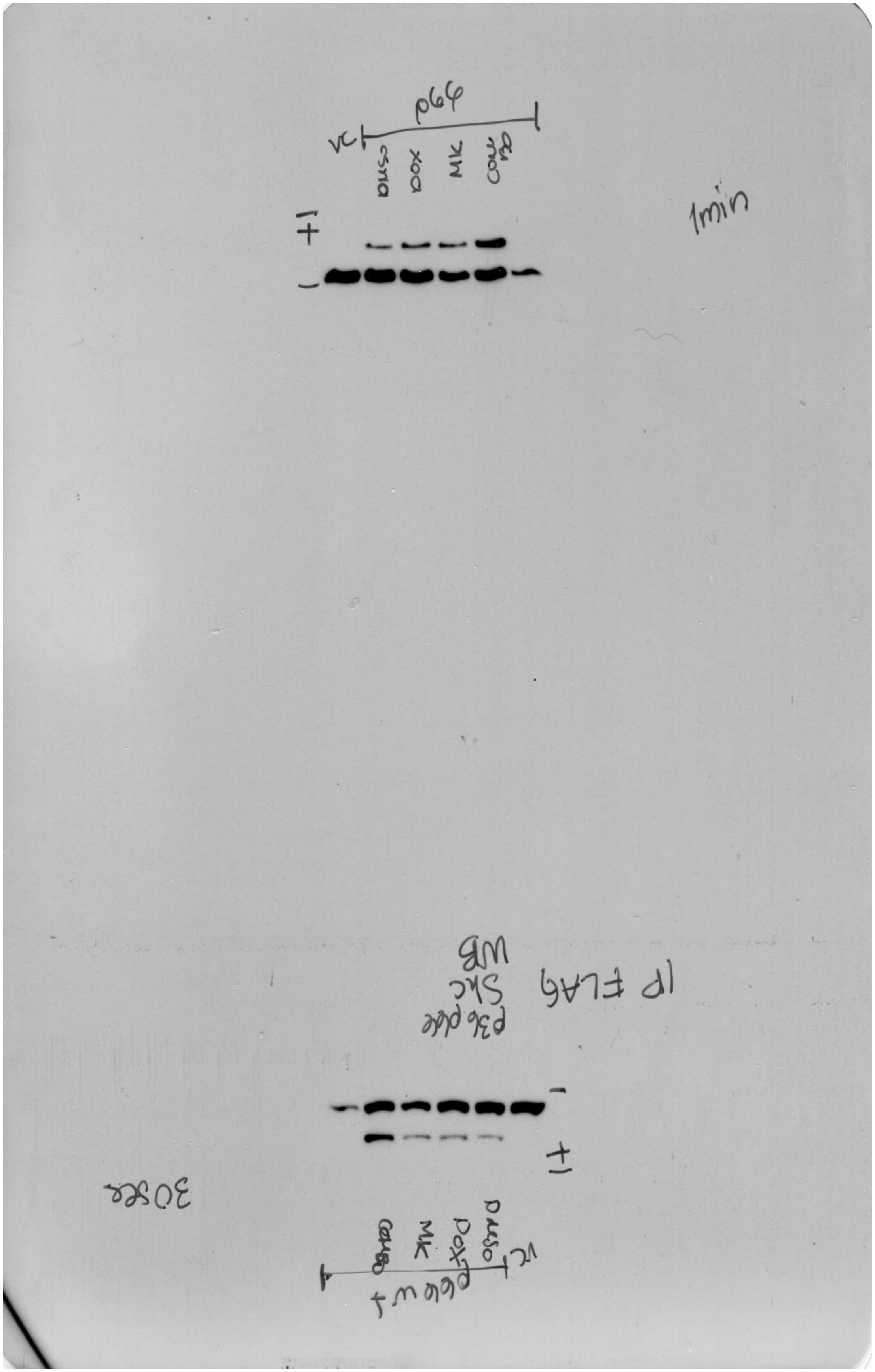

Top left part of the film was used for Figure 4A IP FLAG Western Blot ShcA. The + mark represents 75kDa of the protein ladder.

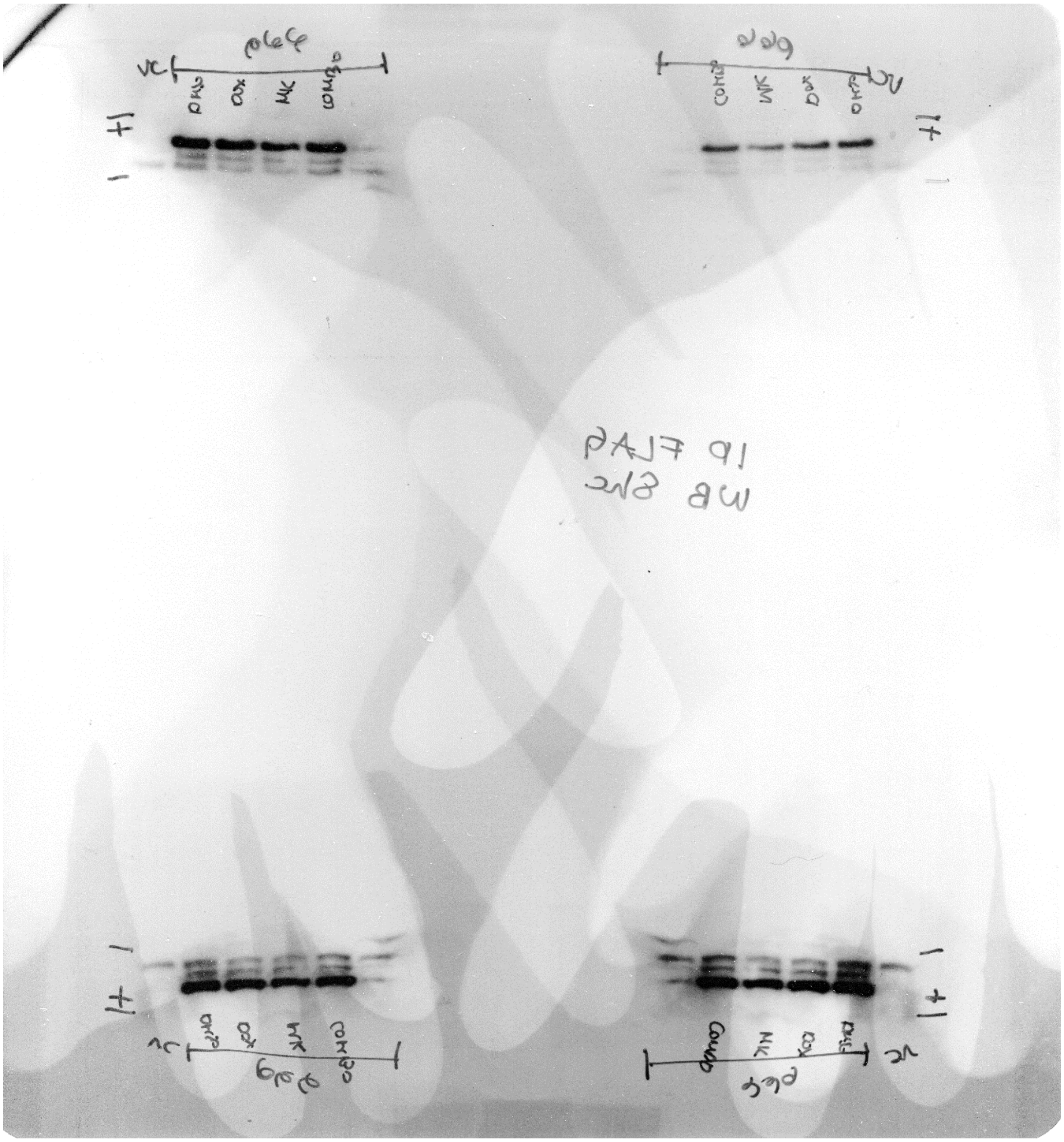

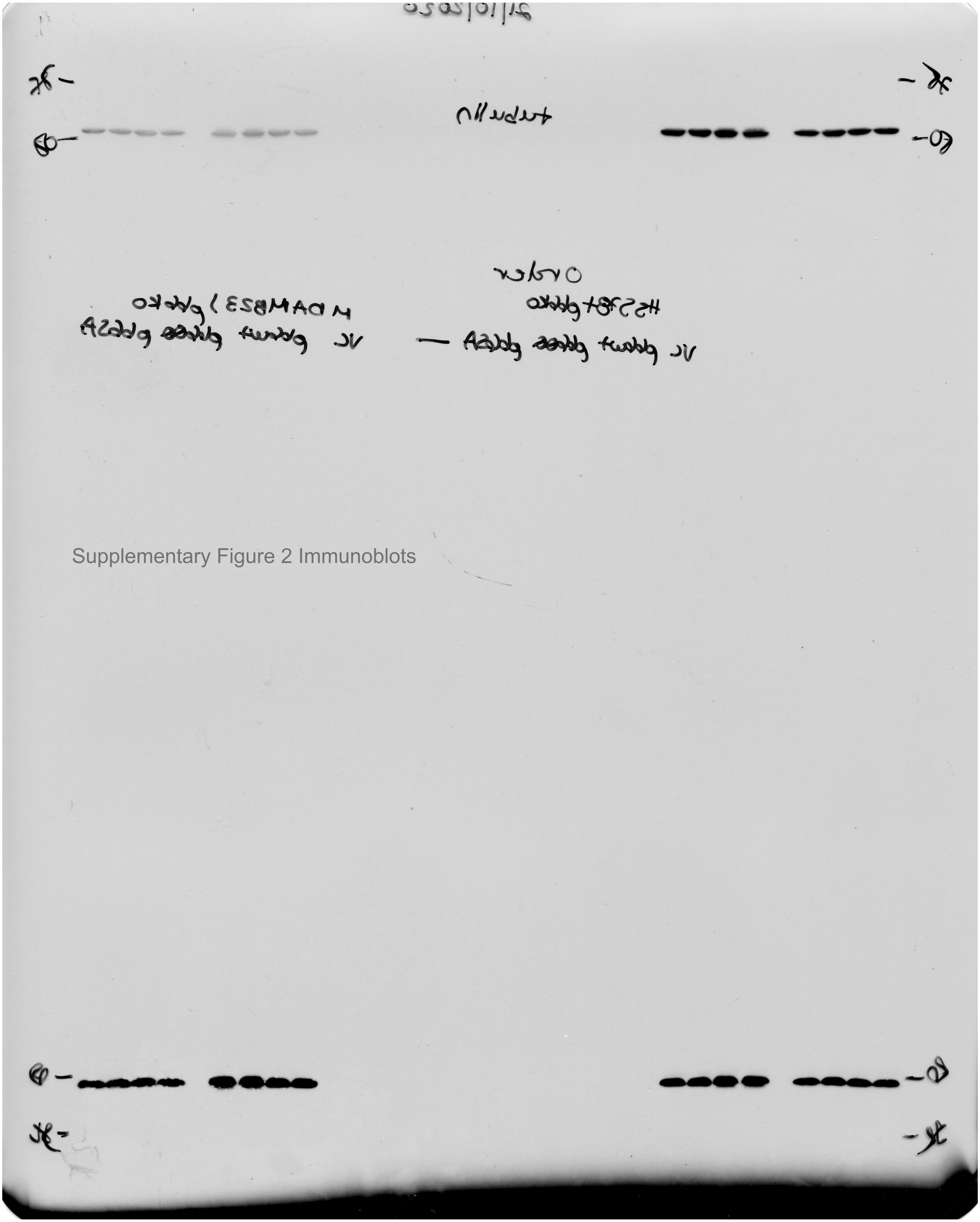

Supplementary Figure 2 Immunoblots

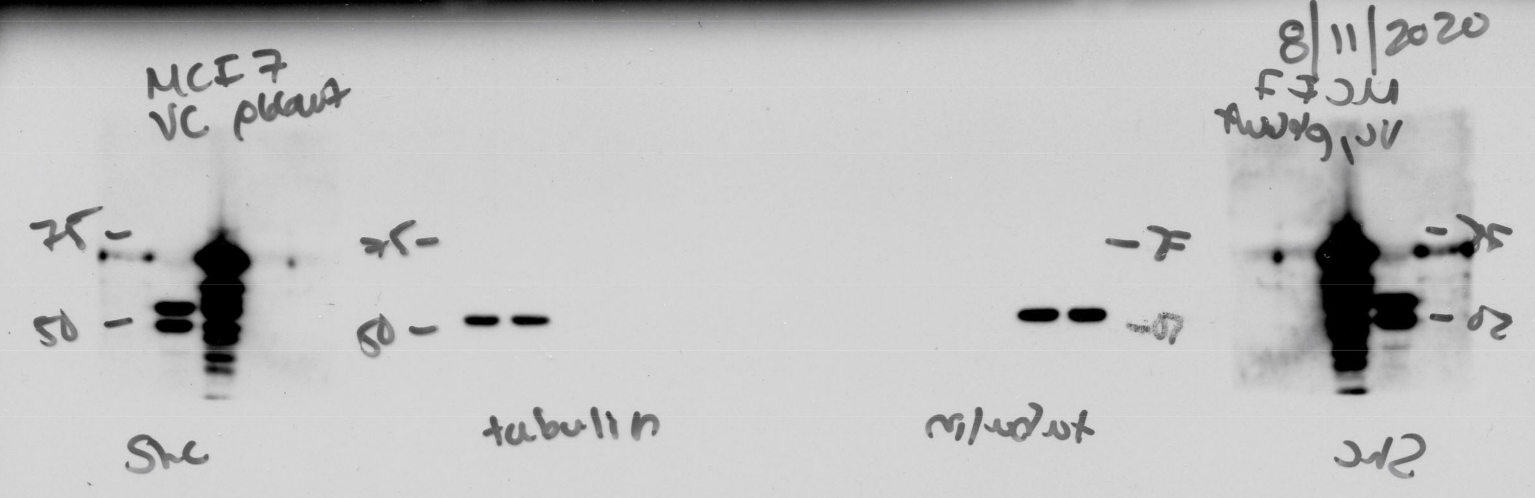

Supplementary Figure 3

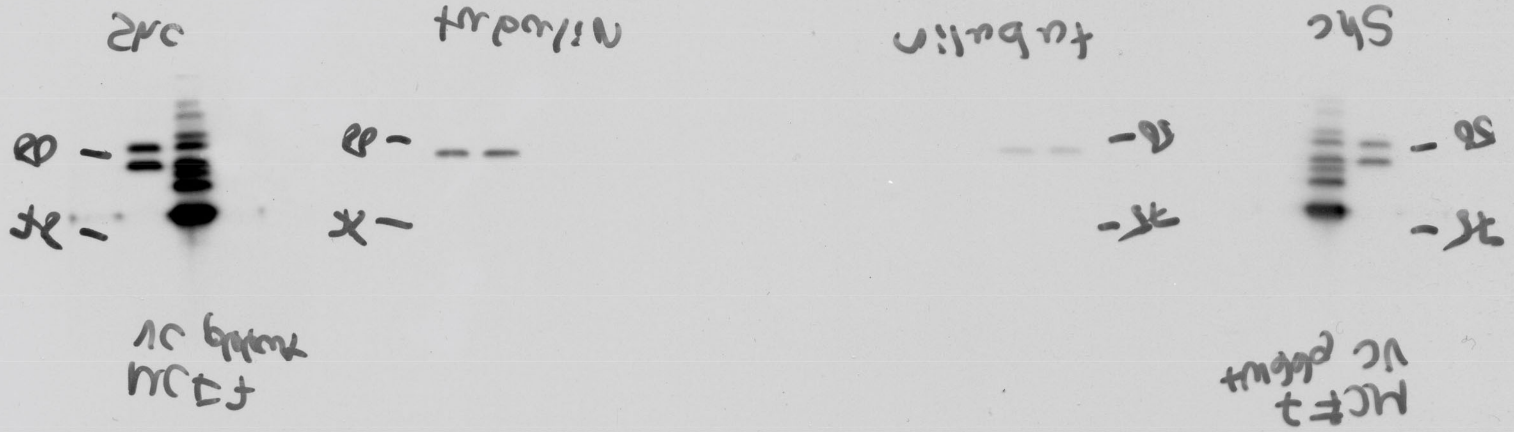

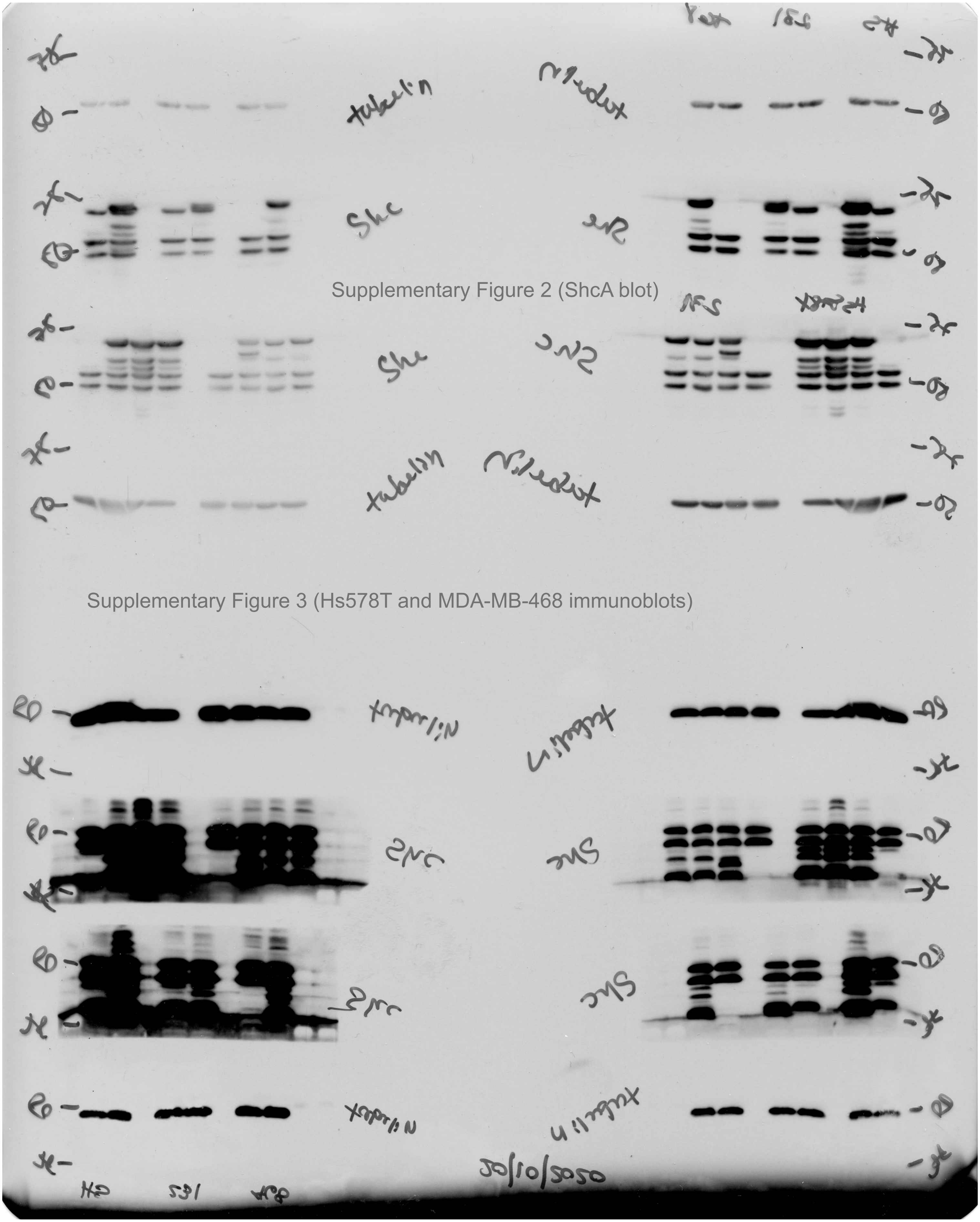

Supplementary Figure 2 (ShcA blot)

Supplementary Figure 3 (Hs578T and MDA-MB-468 immunoblots)

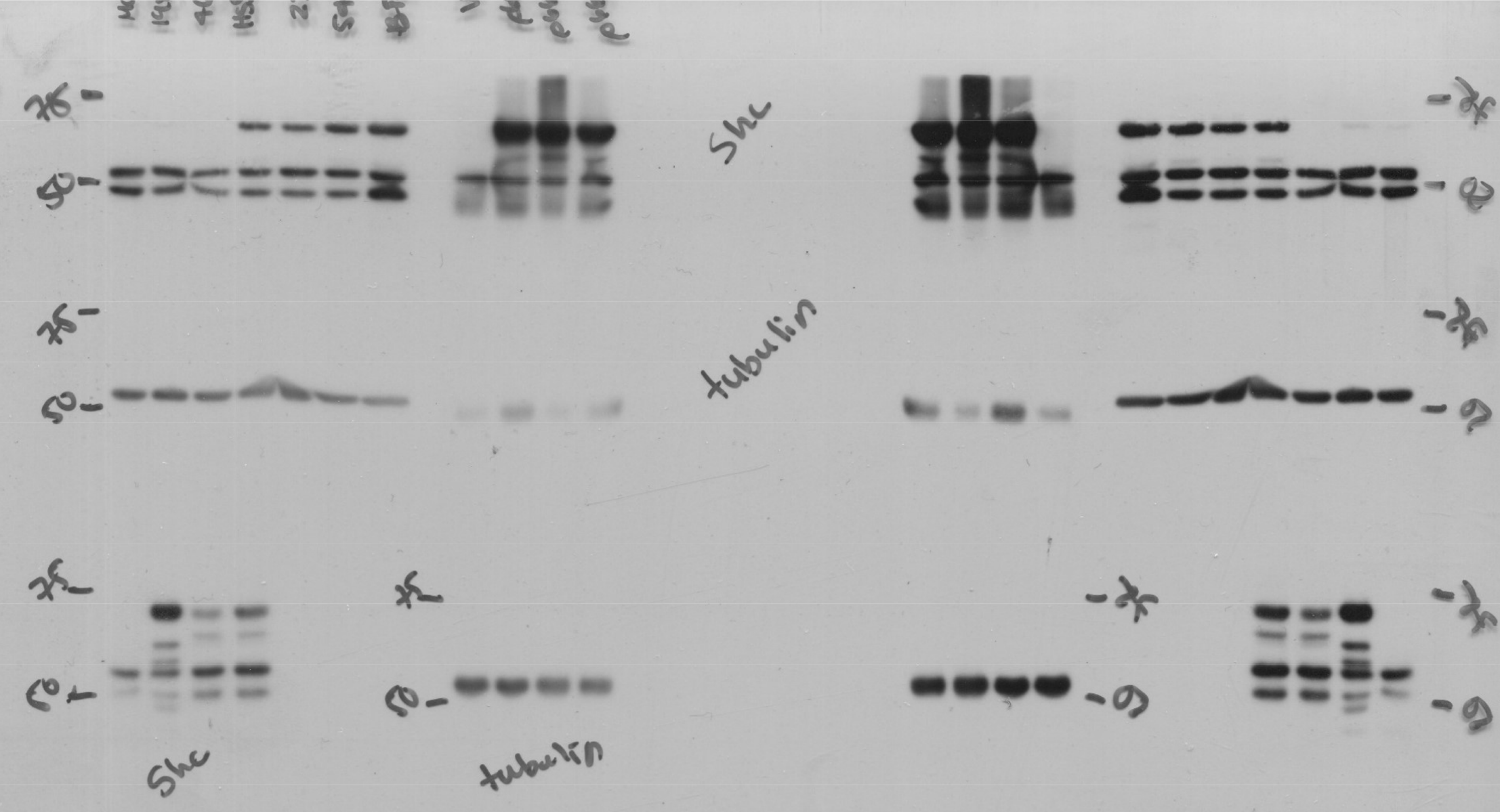

Supplmentary Figure 4

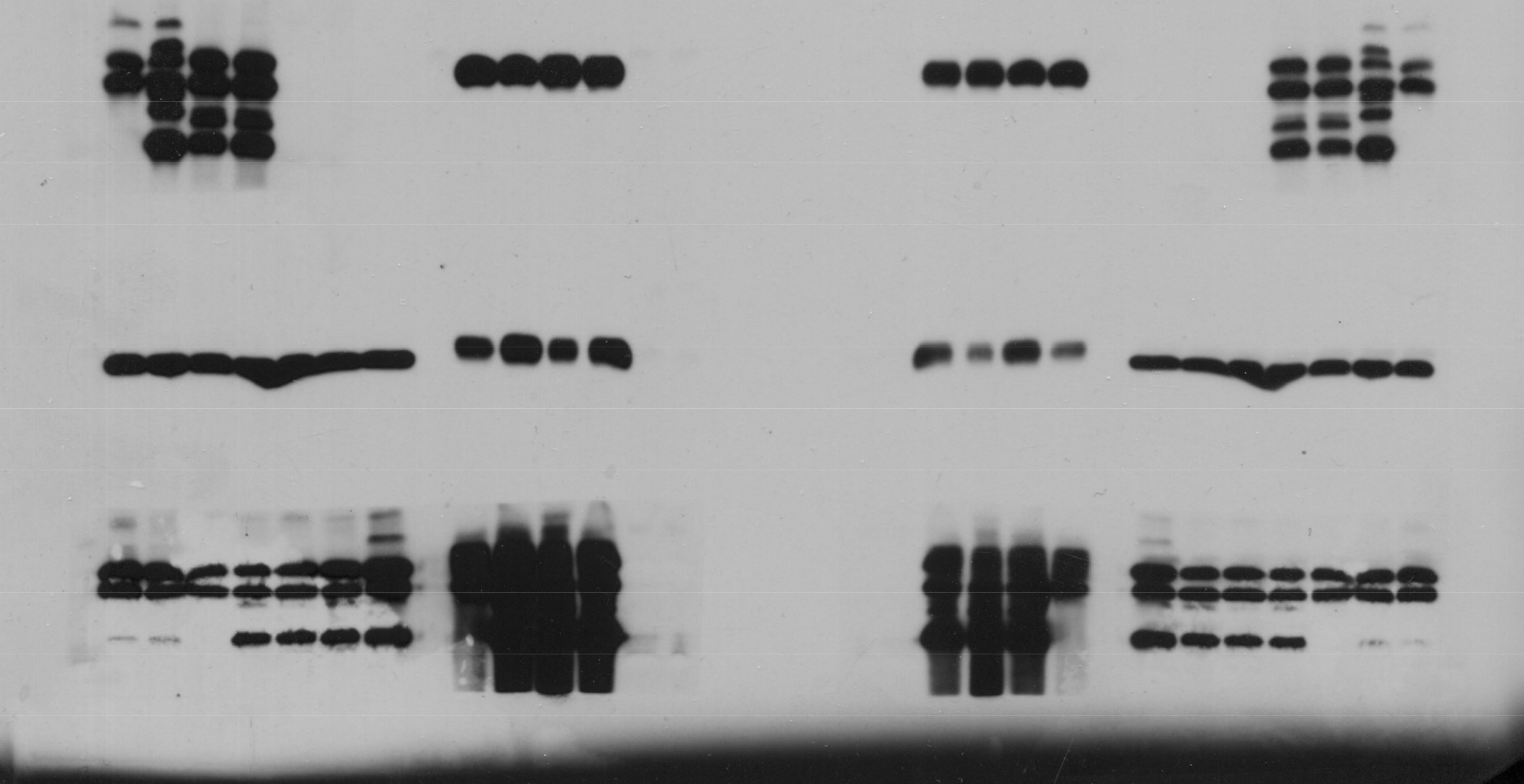

Supplement: Supplemental data [file jciinsight-6-138382-s114.pdf]
